# Central nervous system lymphatic network: from the maintenance of brain homeostasis to emerging therapeutic perspectives in neurodegenerative diseases

**DOI:** 10.1186/s40035-026-00579-9

**Published:** 2026-09-01

**Authors:** Shixin Ding, Jiguang Yang, Ze Wang, Ruiliang Bai, Kuiying Yin, Lei Jin, Maximilian Kueckelhaus, Sven G. Meuth, Ming Xiao, Gang Hu

**Affiliations:** 1 Jiangsu Key Laboratory of Neurodegeneration, Nanjing Medical University, Nanjing, 211166 China; 2 Department of Geriatric Neurology, Affiliated Nanjing Brain Hospital, Nanjing Medical University, Nanjing, 210029 China; 3 Wuxi People’s Hospital, Wuxi Medical Center, Nanjing Medical University, Wuxi, 214000 China; 4 School of Brain Science and Brain Medicine & Liangzhu Laboratory, Zhejiang University School of Medicine, Hangzhou, 311121 China; 5 Link Sense Laboratory, Nanjing Research Institute of Electronic Technology, Nanjing, 210039 China; 6 Department of Plastic and Reconstructive Surgery, Institute of Musculoskeletal Medicine, University Hospital Muenster, 48157 Muenster, Germany; 7 Department of Neurology, University Hospital Muenster, 48157 Muenster, Germany

**Keywords:** Central lymphatic network, Neurodegenerative diseases, Brain homeostasis, Immunosurveillance, Therapeutic targets

## Abstract

The brain was long been regarded as an immune-privileged organ with a lack of lymphatic drainage, partly because of the absence of parenchymal lymphatic vessels. This long-held doctrine has been challenged by recent advancements in central lymphatic vascular biology. The central drainage network, which consists of the glymphatic system, meningeal lymphatic vessels, and cranial perineural drainage pathways, plays a crucial role in the clearance of metabolic brain waste and the maintenance of brain homeostasis. Furthermore, the meninges and brain perivascular spaces are rich in immune cells that regulate neuronal activities and diverse behaviors through secretion of cytokines. Dysfunction of the central lymphatic network is widely observed in natural aging and neurodegenerative disorders, which is characterized by mislocalization of aquaporin-4 on astrocytic endfeet, impairment of lymphatic valvular integrity, and alterations in the proportion and phenotype of meningeal immune cells. In this review, we provide a comprehensive overview of the anatomical foundations, molecular regulatory mechanisms, and physiological functions of this system. We summarize the pathological roles of the central lymphatic network across neurodegenerative conditions and discuss emerging clinical assessment frameworks based on multimodal imaging and liquid biomarkers. We also highlight promising therapeutic strategies, such as restoring meningeal lymphatic function, targeting glymphatic drainage, and modulating the meningeal immune microenvironment. Elucidating the dynamics of the central lymphatic network will reshape our understanding of brain homeostasis, paving the way for novel diagnostic and therapeutic strategies for neurodegenerative diseases. Future research should focus on translating fundamental findings into clinical applications, thereby accelerating the transition from basic research to clinical practice.

## Background

For decades, the brain has been regarded as a relatively isolated “immune-privileged organ” with limited lymphatic drainage, a paradigm rooted in the absence of parenchymal lymphatic vessels and the presence of the blood–brain barrier (BBB) [1]. Under physiological conditions, peripheral immune cells have extremely limited entry into the central nervous system (CNS), and metabolic waste clearance was historically considered to be attributed to passive diffusion or circulatory washout [1]. Nevertheless, this model is unable to elucidate the active immunosurveillance, dynamic cerebrospinal fluid (CSF)-interstitial fluid (ISF) exchange, and region-specific protein aggregation in neurodegenerative diseases. Advances in animal and human studies have collectively demonstrated that the CNS maintains an extensive lymphatic network that comprises the glymphatic system, the meningeal lymphatic vessels (MLVs), and the auxiliary transcranial routes, actively regulating brain homeostasis and immune crosstalk [2, 3].

Evidence supporting this network came from multiple tiers. In 2012, Maiken Nedergaard's group initially characterized a functional brain draining pathway in mice, highly reliant on the perivascular spaces (PVS), astrocytic endfeet, and aquaporin-4 (AQP4)-mediated water flux [4]. Subsequently, this clearance pathway was designated as the glymphatic system [5]. This discovery significantly advanced understanding of the metabolic waste clearance mechanisms within the brain. Specifically, CSF enters the brain parenchyma through periarterial PVS surrounding penetrating arterioles, where it exchanges solutes with the ISF. The mixed CSF–ISF is then directed toward perivenous spaces, ultimately facilitating the clearance of metabolic waste products from the brain [4]. AQP4 located on astrocytic endfeet plays an essential role in this process by facilitating CSF–ISF exchange, thereby enabling the efficient removal of brain macromolecular waste products [4, 6]. Subsequent imaging evidence confirmed the glymphatic transport in humans. Utilizing magnetic resonance imaging (MRI), Ringstad et al. [7] demonstrated CSF tracer entry into periarterial spaces. Separately, during intraoperative thrombectomy, Raz et al. [8] observed contrast agent uptake into the brain parenchyma, supporting CSF-ISF exchange.

In 2015, research groups led by Kipnis [9] and Alitalo [10] independently identified and characterized MLVs in mice. These vessels are predominantly located along the dura mater adjacent to the superior sagittal, transverse, and sigmoid sinuses. They express canonical lymphatic endothelial markers, including lymphatic vessel endothelial hyaluronan receptor-1 (LYVE-1), prospero homeobox protein 1 (Prox1), vascular endothelial growth factor receptor-3 (VEGFR-3), and podoplanin. MLVs connect to the deep cervical lymph nodes (dCLNs), enabling the drainage of CSF, immune cells, and macromolecules into the peripheral lymphatic system [10]. Ex vivo human anatomical studies have confirmed the existence of dural lymphatic vessels. Gadobutrol-enhanced MRI has been used to further corroborate the existence of this structure in humans and primates and validate its functional correlation with CSF [11].

Subsequent research demonstrated that the CSF can bypass the BBB and exit the brain through the cribriform plate along olfactory nerve pathways, ultimately draining into the dCLNs [12]. This brain–nasal route serves as a critical auxiliary pathway for CSF efflux while simultaneously establishing an anatomical basis for intranasal drug delivery to the CNS [13]. The coordinated drainage of MLVs and nasal lymphatic vessels (NLVs) forms a continuous conduit between intracranial fluid compartments and the peripheral immune system. Non-invasive physical neuromodulations, such as repetitive transcranial magnetic stimulation (TMS), can alter the brain fluid drainage dynamics [14]. Chemical stimulation, including intranasal drug administration and nasal perfusion, can modulate the nasal mucosal permeability and thereby enhance drug transport through the olfactory nerve routes into the NLVs [13]. Cumulative evidence indicates that while the brain–nasal route is a well-validated auxiliary CSF outflow pathway in rodents [12], its physiological contribution to the total CSF clearance in humans is likely secondary to that of MLVs [15]. Additionally, lymphatic vessels have been identified in the cornea (under pathological conditions), ciliary body, choroid, and the meningeal coverings of the optic nerve [16]. The glymphatic system has also been described in rodent and human eyes [4, 17]. Amyloid-β (Aβ) in the CSF can be transported into the eye through three major brain–eye routes: the optic nerve sheath–lymphatic pathway, the interaxonal space pathway, and the periarterial pathways [18].

**Fig. 1:**
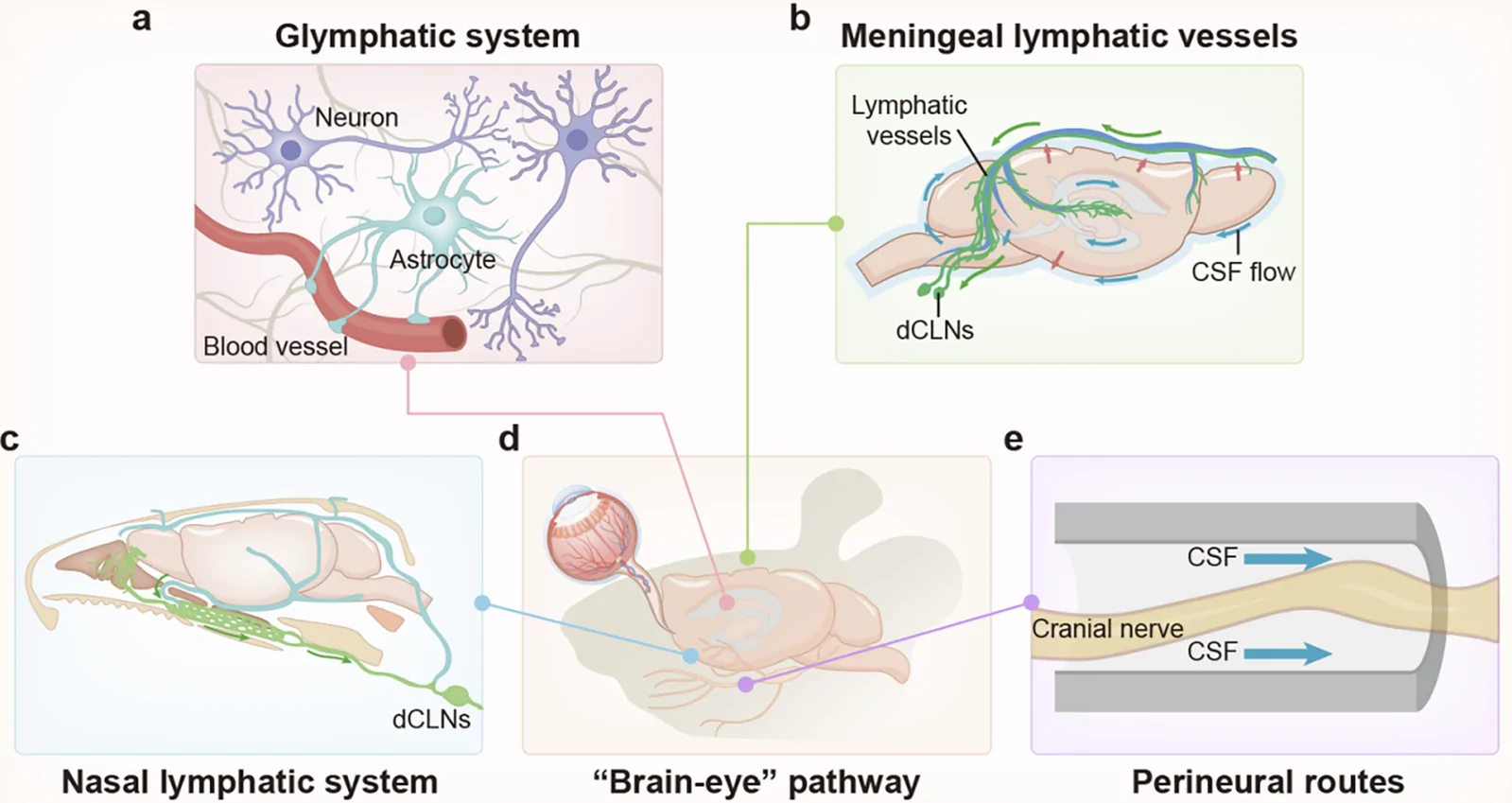
The lymphatic systems associated with the CNS. **a** The glymphatic system, showing the association between astrocytes and brain microvessels. **b** The meningeal lymphatic system, presenting the lymphatic structure of the brain. **c** The nasal lymphatic system, showing the connection between the brain and the NLVs. **d** The brain–eye pathway, depicting the connection between the eyes and the brain. **e** The perineural routes, showing the flow of CSF along the cranial nerves. The structure in **b** was modified from Alves de Lima K, et al. Annu Rev Immunol. 2020;38:597–620 [3] with permission

Taken together, the glymphatic system, MLVs, and accessory pathways such as the brain–nasal and brain–eye routes constitute a multitiered and diversified network for CSF and ISF drainage and immunosurveillance. Here, we propose the term “central lymphatic network” as a conceptual umbrella to unify these pathways, acknowledging that this nomenclature has not yet been standardized in the field (Fig. 1). In the context of aging and neurodegeneration, dysfunction across multiple tiers of this network becomes intertwined, although the onset sequence and the relative contribution of each tier vary across species and disease stages. This review therefore summarizes cross-species and cross-methodological evidence to (1) delineate the anatomical and molecular architecture of each brain draining pathway mentioned above, with explicit attribution of findings to the species in which they were observed, thereby clarifying current knowledge gaps; (2) examine tier‑specific patterns of lymphatic dysfunction in aging, Alzheimer’s disease (AD), and Parkinson’s disease (PD), distinguishing evolutionarily conserved mechanisms from phenomena observed only in preclinical mouse models, and (3) critically evaluate emerging clinical assessment tools, including multimodal MRI and CSF biomarkers, that have been validated for assessing central lymphatic function in humans. Finally, we discuss therapeutic strategies stratified by target network tier, while rigorously acknowledging the ongoing translational gaps between murine preclinical findings and human clinical application.

## Anatomical basis

### Glymphatic system

**Fig. 2:**
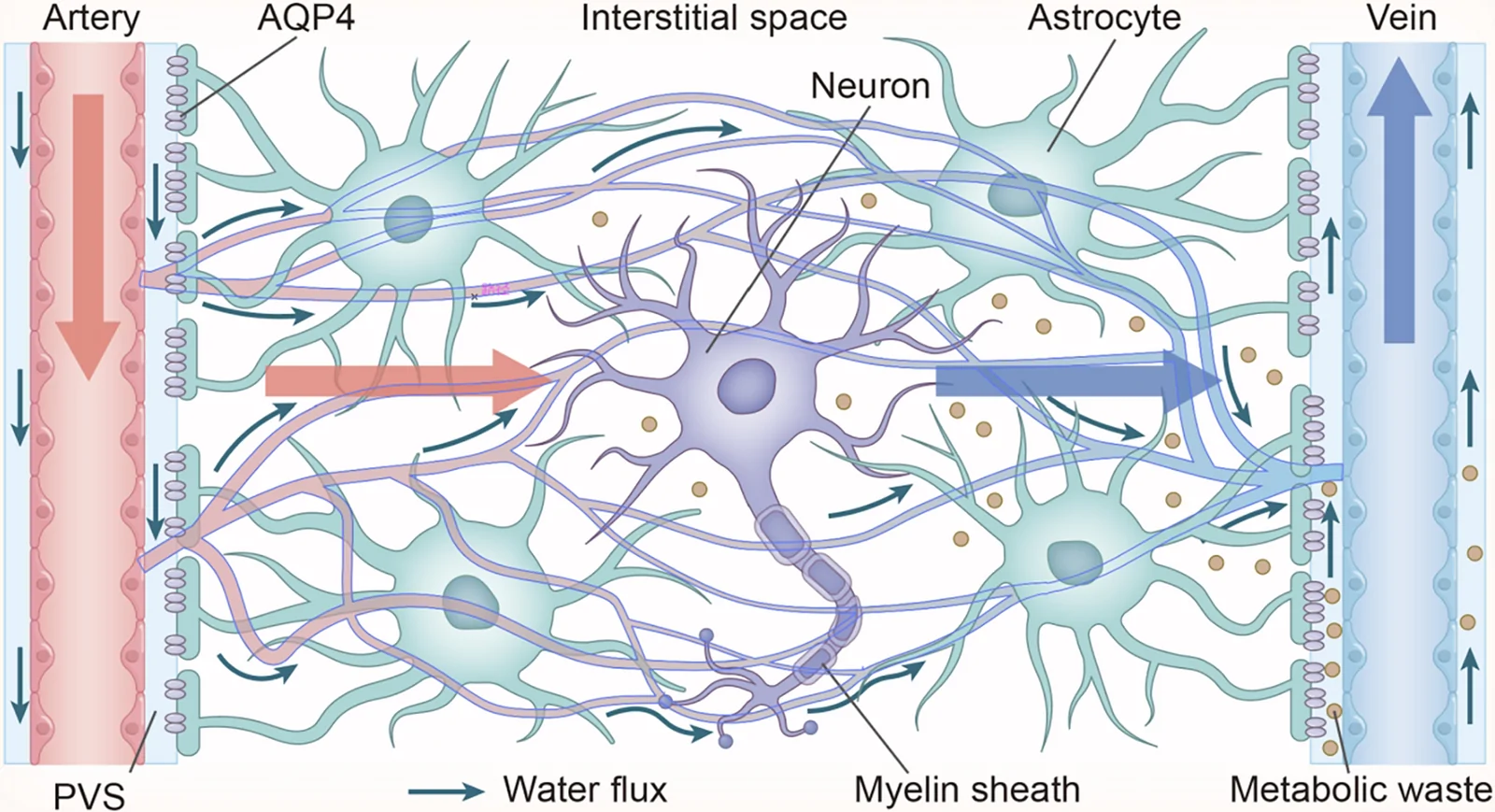
The relevant structures and processes of metabolic waste transport and fluid flow within the glymphatic system mediated by AQP4. Substances carried by arterial blood flow into the PVS through the arterial wall. Here, AQP4 facilitates the transport of water molecules, propelling the fluid to flow within the interstitial space. The fluid, carrying the metabolic waste produced by neurons, follows this flow path and finally converges into the veins

The glymphatic system comprises a functional clearance pathway that critically relies on the coordinated interaction of three dynamic components: (1) periarterial influx pathways formed by PVS, (2) the astrocytic endfoot exchange layer, and (3) perivenous efflux routes [4]. When CSF enters the brain parenchyma from the subarachnoid space, arterial pulsation drives its movement along periarterial PVS. Within the astrocytic “glial tunnels” formed by endfeet, AQP4 channels facilitate rapid CSF–ISF exchange, enabling the solute diffusion-driven clearance of metabolic waste. The fluid then converges along perivenous spaces, forming efflux pathways that ultimately drain through MLVs and dCLNs [4, 9, 10]. Using fluorescence micro-optical sectioning tomography in mice, researchers found that the glymphatic CSF transport exhibits region‑dependent heterogeneity, with preferential ventral entry via regions associated with the circle of Willis (e.g., medulla, pons, hypothalamus) followed by broader but differential distribution, indicating that brain areas near large penetrating arteries or the subarachnoid space serve as preferential perfusion zones [19]. This anatomical heterogeneity reflects differences in arterial pulsatility across brain regions and may underlie the regional variations in metabolic load. Recent human studies using CSF-STREAM (CSF-Selective T₂-prepared REadout with Acceleration and Mobility-encoding) MRI have demonstrated that the CSF motility in the periarterial subarachnoid space is more than twice that observed in the fourth ventricle [20]. This provides evidence for the presence of rapid CSF flow along periarterial pathways in the human brain (Fig. 2).

Unlike the glymphatic system, the intraparenchymal arterial drainage (IPAD) model proposes a distinct anatomical pathway for waste clearance in the CNS [21]. IPAD posits that soluble metabolites are cleared within arterial basement membranes via pulsation- or smooth muscle contraction-driven fluid movement [22]. In contrast, the glymphatic system locates clearance extramurally within perivascular Virchow‑Robin spaces, where AQP4 channels facilitate rapid CSF–ISF exchange [4]. These two routes are largely non‑overlapping anatomically: IPAD operates intramurally, while glymphatic transport operates extramurally [21]. However, tracer studies have shown that the CSF‑borne tracers enter along the pial‑glial basement membranes and later exit along smooth muscle cell basement membranes of the same artery, suggesting that the two routes may be spatially segregated but sequentially coupled rather than strictly independent [23]. Rather than treating them as mutually exclusive theories, emerging evidence suggests that they may function as disease‑specific subsystems [23]. IPAD dysfunction occurs predominantly in cerebral amyloid angiopathy (CAA), where Aβ deposits within arterial walls directly implicate basement membrane clearance failure [24]. Conversely, glymphatic impairment governs parenchymal Aβ and tau accumulation, which correlates with perivascular AQP4 mislocalization [25]. Mechanistically, IPAD efficiency depends on arterial wall elasticity and pulsatility—factors compromised in aging and hypertension—whereas glymphatic function relies on astrocytic AQP4 polarization [22, 25]. Thus, the pathological context may determine which pathway is initially compromised: CAA preferentially disrupts IPAD, while AD and tauopathies primarily impair glymphatic transport. This "dual-track" framework is supported by cross-species evidence. However, the glymphatic concept itself remains a subject of ongoing scientific debate, with current human evidence largely derived from invasive contrast-enhanced MRI, which has inherent limitations [26]. Consequently, whether IPAD and glymphatic clearance represent independent, sequential, or redundant pathways remains an unresolved query, pending technological progress in human imaging.

### Meningeal lymphatic system

**Fig. 3:**
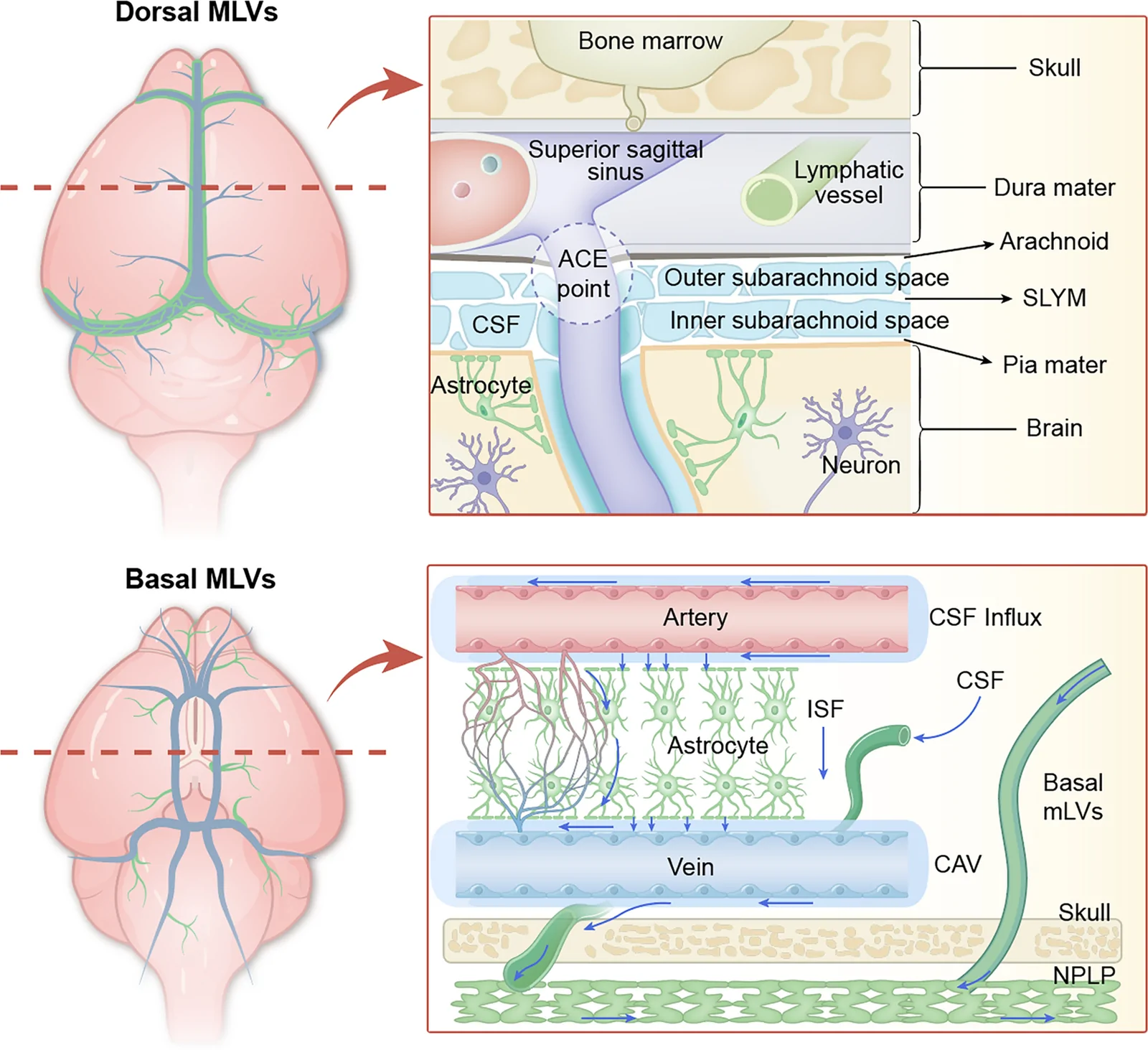
The anatomical structure of meningeal lymphatics is divided into two parts: dorsal MLVs and basal MLVs. CAV: cavernous sinus. NPLP: nasopharyngeal lymphatic plexus. The structure in basal MLVs was modified from Zhang Q, et al. Signal Transduct Target Ther. 2025;10(1):142 [2] with permission

MLVs can be broadly divided into dorsal and basal subdivisions, each with distinct anatomical and functional characteristics [9, 27]. The dorsal MLVs in rodents are predominantly clustered within dural folds surrounding the superior sagittal and transverse sinuses, rather than forming widely distributed networks [9]. They are morphologically less mature, lacking typical lymphatic valves and smooth muscle cell coverage, and exhibit a characteristic “zipper-like” pattern of endothelial junctions with structural discontinuity [28]. The arachnoid cuff exit (ACE) has been identified as a critical interface for fluid and molecular exchange between the CNS and the dural compartment, highlighting a potential contribution of dorsal MLVs to CSF drainage [29]. The basal MLVs in mice have been comprehensively characterized by the Koh group in 2019 [27]. Distributed along the petrosquamosal and sigmoid sinuses, basal MLVs display larger diameters and richer capillary branching compared with the dorsal counterparts [27]. Their terminal segments consist of typical oak leaf-shaped lymphatic endothelial cells equipped with functional valves, enabling efficient unidirectional fluid transport [27]. Notably, Jacob et al. [30] identified an expanded anterior meningeal lymphatic network surrounding the cavernous sinus at the mouse skull base, which interconnects dorsal and basal MLVs and drains the perivenous efflux through skull foramina and fissures into superficial and CLNs, thereby establishing region‑specific drainage routes from the dura mater to the cervical lymphatic system. Basal MLVs exhibit predominantly button‑like endothelial junctions and contain valve structures reminiscent of peripheral collecting lymphatics; however, they display a hybrid phenotype sharing features of both capillary and collecting vessels, with variability in LYVE-1 expression and junctional organization [27]. An additional distinguishing characteristic is the absence of a smooth muscle cell layer in basal MLVs [27]. Collectively, the meningeal lymphatic system demonstrates marked regional heterogeneity and functional complexity. The morphological, junctional, and functional differences between dorsal and basal MLVs (Fig. 3) provide critical insights into CSF drainage and immune regulation within the CNS.

The MLV anatomy and valve function have been further confirmed in non-human primates [31]. In 2025, using dynamic contrast-enhanced MRI, immunofluorescence, and imaging mass cytometry, Albayram et al. [32] provided direct in vivo and ex vivo evidence of organized lymphatic structures along the ventral dura, filling a key gap in the anatomical map of human ventral meningeal lymphatics. Nevertheless, unlike in mice, the overall anatomical characteristics of human dural lymphatics have not been fully established. This is partly due to the significantly larger size of human meningeal specimens compared to those of mice; additionally, the detection of lymphatic endothelial markers is highly susceptible to tissue processing methods, with standard paraformaldehyde fixation often yielding negative results [33]. In vivo imaging of human dural lymphatics also remains challenging. The typical caliber of the human MLVs, ranging from 20 to 100 μm, is an order of magnitude smaller than the spatial resolution of standard MRI, which is approximately 1 mm. Functional monitoring is even challenging. For example, dynamic contrast-enhanced MRI (DCE-MRI)-based attempts also face major limitations due to inadequate spatiotemporal resolution [34]. Currently, even though 7 T MRI can visualize the outline of the meningeal lymphatic network [35], it remains insufficient for fine structural analysis of individual lymphatic vessels. Due to the constraints of skull anatomy, other transcranial optical imaging or acoustic imaging techniques remain in an immature stage of development for detecting human dural lymphatics [36]. Collectively, noninvasive comprehensive detection of structural features of human MLVs, and real-time, precise monitoring of their lymphatic drainage capacity, represent critical technical challenges that require urgent resolution.

While the structural and functional features of MLVs, as outlined above, are broadly supported by converging evidence, a novel putative meningeal structure reported in 2023 remains the subject of ongoing anatomical debate. Nedergaard’s group identified an additional meningeal layer, termed the subarachnoid lymphatic-like membrane (SLYM), and proposed that this Prox1-positive, lymphatic-like mesothelial membrane subdivides the subarachnoid space [37]. However, independent high-resolution molecular mapping of the adult mouse leptomeninges localized Prox1⁺ cells—the reported SLYM marker—precisely within the inner arachnoid/reticular arachnoid, a structure long established in classical neuroanatomy [38]. Further studies are warranted to exclude the possibility that the SLYM might represent a functionally specialized subdomain of the known arachnoid microarchitecture, rather than a discrete anatomical stratum. Accordingly, the proposed barrier and immunological functions of the SLYM require ultrastructural validation in human meningeal tissue before being incorporated into clinical models of CNS lymphatic pathophysiology.

### Nasopharyngeal lymphatic network

The cribriform plate is a porous structure of the ethmoid bone at the skull base, inferiorly adjacent to the frontal lobe and olfactory bulb, containing multiple foramina through which olfactory nerve fibers traverse [39]. The nasal mucosa attached beneath the cribriform plate is richly supplied with capillary lymphatic vessels [39], which form dorsal connections with the MLVs of the supranasal sinus via their rostral projections and establish ventral anastomoses with MLVs surrounding the cavernous sinus [30]. In animal models, these lymphatic vessels form dorsal connections with MLVs of the supranasal sinus and ventral anastomoses with MLVs surrounding the cavernous sinus [30]. In humans, there is currently no direct anatomical evidence that lymphatic vessels cross the cribriform plate to extend into the nasal cavity. In contrast, in various animal models—including mice, rats, lambs, and non-human primates—direct anatomical evidence does support the presence of lymphatic vessels traversing the cribriform plate alongside olfactory nerve bundles, establishing structural connections with nasal submucosal lymphatics. These studies suggest that fluid exchange between the CNS and the nasal lymphatic network likely occurs through the perineural spaces surrounding olfactory nerve bundles, forming a distinct "brain–nasal" lymphatic communication route.

The NLVs are located within the deeper layers of the nasal mucosa and are widely distributed across the olfactory epithelium and the nasal septum [39, 40]. In addition, the lymphatics of the anterior nasal region (including the nasal vestibule and the anterior portion of the nasal septum) primarily drain the superficial nasal mucosa, with lymph flow directed toward the submandibular and submental lymph nodes [40].

In recent years, advances in imaging technologies have led to significant breakthroughs in the characterization of the nasopharyngeal lymphatic network. In 2024, the group led by Koh identified a densely distributed lymphatic network within the nasopharyngeal mucosa in lymphatic-specific fluorescent reporter mice (Prox1-GFP), using in vivo imaging and tissue-clearing techniques, and validated it in non-human primates [12]. Termed the nasopharyngeal lymphatic plexus (NPLP), this structure displays a distinctive “inverted saddle” morphology and exhibits several remarkable anatomical features. First, the plexus is filled with irregularly shaped valves that ensure unidirectional lymph flow. Second, the lymphangions within the NPLP are short and lack smooth muscle coverage, rendering the vessels incapable of actively pumping lymph through rhythmic contraction [12]. These characteristics distinguish the NPLP from both initial lymphatic capillaries, which primarily mediate fluid absorption, and collecting lymphatic vessels, which are characterized by longer lymphangions, smooth muscle layers, and semilunar valves that enable active lymph propulsion. Instead, the NPLP displays features consistent with pre-collecting lymphatic vessels, suggesting that it may play a crucial role in facilitating lymphatic communication along the brain–nasal axis.

While rodent tracer studies robustly demonstrate nasal drainage, direct quantitative evidence for its physiological dominance in humans remains highly limited and inferential. Human evidence is constrained to: (1) MRI-based methods that can quantify CSF velocity or mobility but do not provide volumetric flux in deep perivascular spaces [20]; and (2) postmortem lymphatic mapping, which confirms anatomical presence but not functional capacity [33, 41]. Several lines of evidence support the hierarchical primacy of the dural lymphatic system—particularly basal MLVs—over the nasal route for physiological CSF clearance [42]. First, dural MLVs exhibit far greater structural maturity (functional valves, smooth muscle coverage) and greater functional capacity for macromolecular clearance in both rodents and non-human primates [42, 43]. Second, surgical ablation of dural MLVs in mice causes profound CSF accumulation and neuroinflammation, effects not replicated by isolated nasal pathway disruption [42]. Third, in comparative functional imaging studies, DCE-MRI revealed significantly higher CSF uptake and drainage efficiency in basal MLVs compared with dorsal MLVs in mice, and human ex vivo dural tissue analysis demonstrated denser LYVE-1⁺/PROX1⁺ vessel networks in basal regions versus dorsal dura [42, 43]. Taken together, while the brain–nasal route may serve as a critical auxiliary outflow or pathological bypass, the dural lymphatic system constitutes the primary, high-capacity conduit for CSF and solute drainage under physiological homeostasis. Therapeutic strategies targeting impaired CSF clearance may prioritize restoring dural lymphatic integrity, with nasal routes considered as secondary targets only for specific clinical contexts.

### Ocular lymphatic vessels

**Fig. 4:**
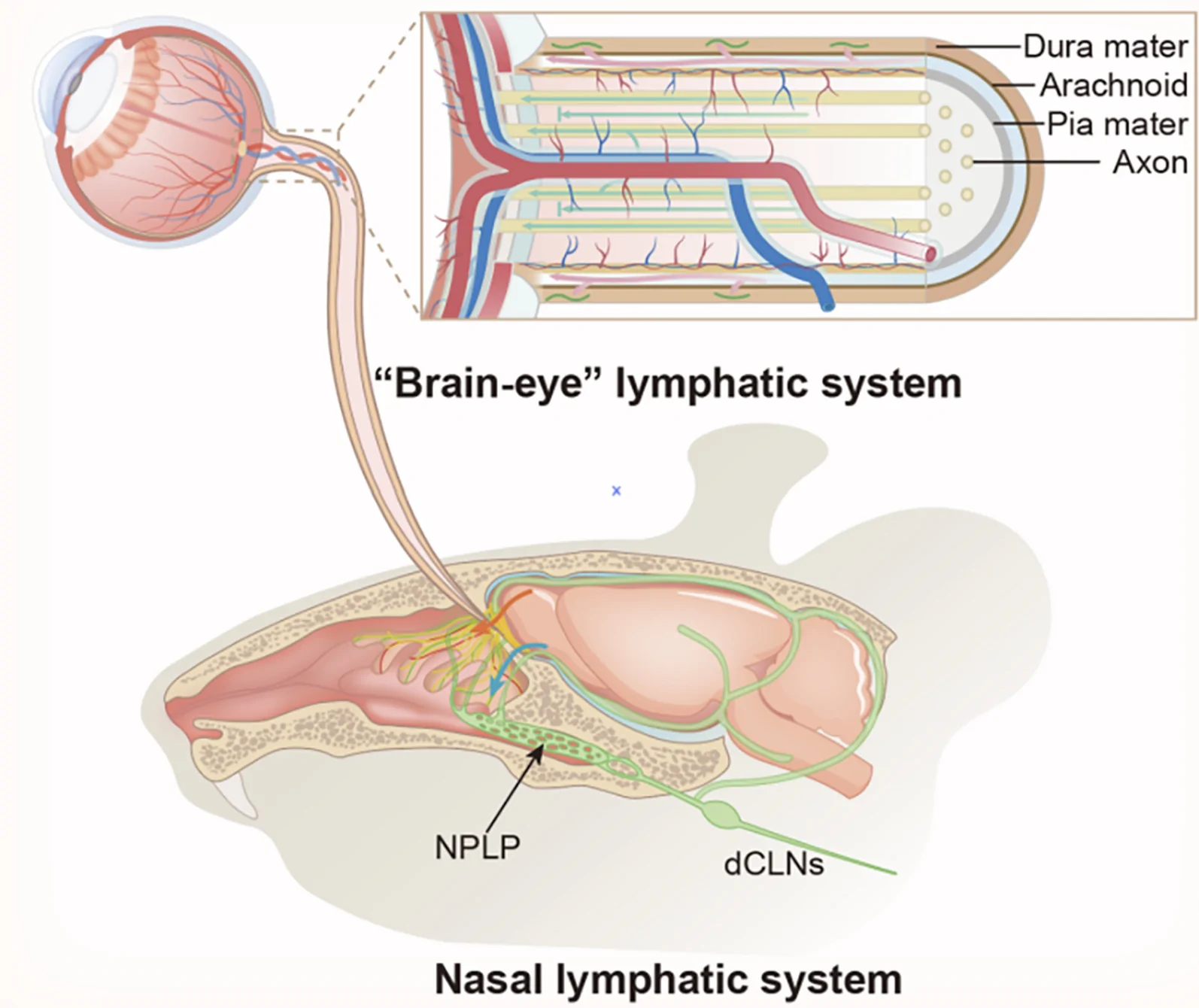
The ocular lymphatic drainage pathway and nasal lymphatic drainage pathway. Ocular lymphatic drainage pathway (upper panel): CSF can enter the optic nerve parenchyma through the PVS around the penetrant small blood vessels, and further flow out of the cranial cavity via the MLVs around the optic nerve. Nasal lymphatic drainage pathway (lower panel): CSF enters the NLVs through the perineural spaces of the olfactory nerves at the cribriform foramina and finally drains into the dCLNs. The structure in ocular lymphatic drainage pathway was modified from Cao Q, et al. J Exp Med. 2024;221(11):e20240386 [18] with permission

Traditionally, fluid outflow from the anterior segment of the eye has been attributed primarily to the trabecular meshwork and the uveoscleral pathways, which are located near the lens and iris [44]. However, recent studies have identified lymphatic vessels expressing canonical lymphatic markers in the limbus, conjunctiva, optic nerve sheath, and other ocular tissues, offering new perspectives on ocular fluid clearance mechanisms [45]. The cornea is a transparent avascular connective tissue that plays essential roles in refractive function and immune homeostasis [46]. Under physiological conditions, lymphatic vessels in the cornea are confined to the limbal region, where they connect with the conjunctival and periocular lymphatic networks [46]. This restricted distribution helps maintain corneal transparency while contributing to local immune regulation [46]. In contrast, the conjunctiva, a mucosal tissue covering the inner eyelids and the anterior surface of the eyeball, contains the richest lymphatic network in the eye [47, 48]. It can be divided into bulbar, palpebral, and fornical conjunctiva, and its lymphatic system consists of superficial fine lymphatic capillaries and deeper, larger lymphatic vessels [48]. Lymphatic structures within the optic nerve sheath are regarded as an important drainage route linking the eye and the CNS [49]. Experimental studies have demonstrated that, following tracer injection into the subarachnoid space, CSF tracers enter and travel along the optic nerve and are subsequently cleared via periocular tissues and cervical lymph nodes, providing functional evidence for an optic nerve-associated glymphatic/lymphatic drainage pathway [50–52]. Canonical lymphatic vessels have also been identified in the lacrimal gland. Histochemical and electron microscopy analyses, together with podoplanin immunolabeling, indicate that these lymphatics may participate in tear metabolism and local immune modulation [53]. Schlemm’s canal, a unique structure that exhibits both vascular and lymphatic characteristics, plays a crucial role in regulating intraocular pressure [45]. In cooperation with the trabecular meshwork, it mediates aqueous humor outflow and helps remove bacteria and metabolic waste, thereby maintaining ocular fluid homeostasis [45]. In addition, several studies have proposed the existence of a glymphatic system within the retina [47, 54]. The abundant astrocytes and AQP4-enriched endfeet distributed throughout the retina and optic nerve provide a plausible structural basis for such a system [55]. Nevertheless, the precise functions of a potential retinal glymphatic system and its relationship with other ocular and CNS clearance pathways remain to be fully elucidated. Whether lymphatic vessels exist in the extraocular muscles and choroid remains a matter of debate. Some studies have detected lymphatic structures in the connective tissue of all extraocular muscles [56], whereas others observed only sparse lymphatic elements near the anterior portion of the levator palpebrae superioris muscle [53]. Similarly, although lymphatic channels or pre-lymphatic structures have been reported in the choroid, these lack classical lymphatic markers and definitive anatomical connections to downstream LNs [57, 58]. Furthermore, single-cell sequencing combined with lymphatic lineage-tracing mouse models has confirmed that the ciliary body lacks lymphatic vessels under physiological conditions [59] (Fig. 4).

## Spatial organization of brain clearance: the nearest‑exit principle and the skull as a drainage hub

A recent study by Chayama et al. circumvented this limitation of conventional CSF tracer approaches by developing a neuron‑specific ZsGreen reporter mouse (Syn‑ZsG) to trace endogenously produced neuronal proteins, revealing four unexpected principles that substantially revise our understanding of the central lymphatic network [60]. First, neuronal proteins follow clearance routes—dorsal dura mater, skull bone marrow, nasal mucosa, and cervical lymph nodes—that are largely missed by conventional CSF tracers, suggesting that previous estimates of cranial lymphatic function may require revision [60]. Second, brain waste clearance obeys a “nearest‑exit principle”: different brain regions preferentially clear proteins through their anatomically closest boundary tissues (e.g., cortical proteins drain to the overlying skull, whereas striatal proteins rely more on the olfactory route), providing a mechanistic framework for region‑selective vulnerability in neurodegeneration [60]. Third, acute neuroinflammation and chronic amyloid pathology impair clearance through distinct mechanisms—vascular shunting versus parenchymal trapping combined with efflux failure—implying that therapeutic strategies to restore brain waste removal must be tailored to the underlying disease [60]. Collectively, these findings transform the central lymphatic network from a set of passive conduits into a spatially organized, dynamically regulated system, adding a topological dimension to its anatomical architecture and providing a mechanistic rationale for disease‑stratified therapeutic interventions.

## Molecular basis

### AQP4

A growing body of evidence indicates that glymphatic transport critically depends on the highly selective perivascular expression of AQP4 in the brain microvasculature. AQP4 is the most abundantly expressed AQPs in the CNS and plays a central role in facilitating water movement within the brain, serving as an essential component of the glymphatic system [61]. AQP4 exhibits extraordinarily high water permeability and is polarized to the perivascular endfeet of astrocytes, anchored by the dystrophin-associated complex via α-syntrophin [62–64]. This unique spatial distribution and its tight regulation by circadian rhythms, mechanical stress, and isoform-specific assembly (M1/M23 ratio), constitute the definitive molecular basis for directional CSF–ISF exchange, perivenous solute efflux, and glymphatic clearance efficiency [64–71]. Critically, loss of this polarized localization—not mere reduction in total protein—is the primary driver of glymphatic dysfunction across aging and neurodegeneration [65, 66, 72, 73]; therefore, all subsequent discussions of AQP4 in disease contexts or therapeutic strategies refer exclusively to this mechanistic framework established herein.

In the mouse brain, AQP4 exists predominantly in two isoforms, M1 and M23 [67]. While both isoforms exhibit similar water-transport capacities, they differ markedly in their aggregation properties and subcellular localization within astrocytes [67]. Notably, M23 is highly enriched in the perivascular endfeet of astrocytes [67, 68], and its localization depends on the dystrophin-associated complex, whose core components include dystrobrevin (DTNA), dystrophin (DMD), dystroglycan (DAG1), and AQP4—all strongly expressed in astrocytes [69]. The transcription levels of *DAG1*, *DTNA*, and *AQP4* are significantly higher during the daytime (the sleep period for mice) than at night, correlating closely with fluctuations in glymphatic influx [70, 74]. Although absolute levels of M1 and M23 do not vary substantially across the circadian cycle, the M1/M23 ratio decreases during the daytime, suggesting preferential perivascular localization of AQP4 and enhanced glymphatic activity [70]. These findings indicate that AQP4 expression and assembly are under circadian regulation, offering potential targets for modulating glymphatic transport.

In addition to its role in CSF–ISF exchange, AQP4-M23 also regulates astrocytic process motility and glutamate-dependent synaptic transmission. Using live imaging of astrocytic process motility and neuronal calcium transients, recent studies have revealed a key role of AQP4‑M23 in these processes [67, 75]. In patients with neuromyelitis optica (NMO), anti-AQP4 antibodies (NMO-IgG) disrupt the membrane distribution of AQP4-M23, thereby impairing astrocyte–neuron communication [71]. These findings advance our understanding of tripartite synapse regulation.

### Vascular endothelial growth factor (VEGF)-C/D–VEGFR-3

VEGF-C and VEGF-D are key members of the VEGF family that regulate the survival, proliferation, migration, and differentiation of lymphatic endothelial cells by binding to their cognate receptor VEGFR-3, encoded by the *FLT4* gene. Through these actions, they play essential roles in lymphangiogenesis and the maintenance of lymphatic vessel integrity [76]. VEGFR-3 is predominantly expressed on lymphatic endothelial cells, where its activation triggers downstream phosphatidylinositol 3-kinase (PI3K)/protein kinase B (AKT) and mitogen-activated protein kinase (MAPK)/extracellular signal-regulated kinase (ERK) signaling pathways, promoting lymphatic endothelial cell proliferation, motility, and lymphatic branching morphogenesis [77]. Under physiological conditions, VEGF-C/D binding to VEGFR-3 activates the PI3K/AKT and MAPK/ERK cascades, supporting lymphatic endothelial cell survival, migration, and lymphatic network expansion [76]. During embryonic development and tissue homeostasis, VEGFR-3 signaling maintains the structural and functional organization of the lymphatic system, not by markedly increasing lymphatic endothelial cell numbers but by regulating their differentiation, spatial distribution, and vessel patterning [78]. In pathological conditions such as lymphatic malformations and tumor-associated lymphatic metastasis, excessive activation of VEGFR-3 drives aberrant lymphatic endothelial cell proliferation and hyperplastic lymphangiogenesis [79]. Pharmacological inhibition of VEGFR-3 by agents, such as MAZ51, can block lymphatic vessel formation and has been explored as a therapeutic strategy for lymphatic malformations and metastatic cancer [80]. Conversely, strategies that enhance VEGFR-3 signaling, including the use of VEGF-C analogs, may promote lymphatic vessel regeneration [81]; however, their potential impacts on normal tissue homeostasis require careful evaluation.

### Other growth factors and cytokines

Although VEGF-C/VEGFR-3 signaling is indispensable for both developmental and postnatal lymphangiogenesis, several additional growth factors and cytokines have been shown to independently or cooperatively induce lymphatic vessel formation. Ectopic expression of fibroblast growth factor-2 (FGF2) [82], VEGF [83], platelet-derived growth factor [84], epidermal growth factor [85], insulin-like growth factor-1 [86], and hepatocyte growth factor (HGF) [87] can each stimulate lymphangiogenesis in mouse tissues. Notably, inhibition of VEGF-C/D signaling suppresses lymphatic vessel growth induced by FGF2 [82], angiopoietin-1 [88], and HGF [89], suggesting that these growth factors may exert their lymphangiogenic effects through mechanisms that are at least partially dependent on VEGF-C/D. In contrast, ectopic expression of thrombospondin-1 [90] and epithelium–derived factor [91] can inhibit lymphangiogenesis in mouse tissues. Beyond direct actions on lymphatic endothelial cells, some growth factors may also influence lymphangiogenesis through indirect mechanisms. For example, certain growth factors may recruit leukocytes that subsequently produce VEGF-C/D, thereby indirectly promoting lymphatic vessel growth [92]. Another possibility is that these factors induce VEGF-C expression in blood endothelial cells or adjacent smooth muscle cells, which then enhances lymphangiogenesis through paracrine signaling [82]. Collectively, these findings highlight that lymphangiogenesis is a complex biological process governed by a diverse array of growth factors. These factors regulate lymphatic development not only by acting directly on lymphatic endothelial cells but also through multiple indirect pathways, including modulation of VEGF-C/D production or release. Such complicated regulatory networks provide important foundational insights into the molecular mechanisms underlying MLV development and postnatal remodeling, which we will discuss in subsequent disease sections.

### Mechanical signals and lymphatic valve

Mechanical signals play a central and non-redundant role in regulating lymphatic flow dynamics, developmental lymphangiogenesis, and lymphatic valve morphogenesis [93]. Vascular stretch triggered by lymph flow increases intracellular calcium to induce vessel contraction, while the resulting fluid shear stress stimulates nitric oxide production and promotes local lymphatic relaxation [93]. Elevated lymph flow also modulates the expression of chemokines and adhesion molecules in lymphatic endothelial cells, enhancing immune cell trafficking and facilitating rapid immune responses [94]. These findings underscore that lymph flow itself is a core regulator of lymphatic endothelial cell function and lymphatic network homeostasis [95]. During mouse embryonic development, lymphatic vessel expansion is tightly associated with peak ISF pressure and lymphatic endothelial cell elongation [96]. Reduced ISF pressure shortens lymphatic endothelial cells, suppresses VEGFR-3 tyrosine phosphorylation, and inhibits lymphatic endothelial cell proliferation; conversely, increased ISF pressure elongates lymphatic endothelial cells, enhances VEGFR-3 phosphorylation, and promotes lymphatic endothelial cell proliferation [96]. Further research demonstrated that β1-integrin is required for lymphatic endothelial cells to mount proliferative responses to changes in fluid volume and cellular stretch, revealing a mechanotransduction-based mode of VEGFR-3 activation that is essential for normal mammalian embryogenesis and fluid homeostasis maintenance [96].

As a mechanically gated cation channel of the Piezo protein family, Piezo1 serves as a key sensor and transducer of mechanical stimuli in the lymphatic system. It can be activated by multiple mechanical cues including shear stress, substrate stiffness, and cyclic pressure, converting these mechanical signals into intracellular electrical responses [97, 98]. By sensing fluid-generated mechanical forces, Piezo1 activates intracellular calcium signaling, downregulates Notch signaling, and promotes lymphatic sprouting and expansion via the VEGFR-3 activation cascade described above [99]. Both genetic and pharmacological activation of Piezo1 markedly enhances lymphangiogenesis and lymphatic function, improving tissue fluid homeostasis [99].

Piezo1 plays a critical role in lymphatic valve development, influencing lymphatic endothelial cell differentiation and valve morphogenesis through the angiopoietin/Tie/Foxo1 signaling axis, thereby ensuring unidirectional lymph flow [100]. Lymphatic valves are essential structures that maintain unidirectional lymph flow from peripheral tissues back to the systemic circulation; morphological abnormalities in these valves cause lymphatic or venous reflux and ultimately lead to lymphedema [101, 102]. Lymphatic valves typically arise at vessel branch points, indicating that lymphatic endothelial cells exposed to distinct flow patterns initiate developmental programs. When human lymphatic endothelial cells are cultured under oscillatory (rather than laminar) shear stress, they upregulate forkhead box C2 (FOXC2), adopt a cuboidal morphology, increase connexin-37 expression, and activate the calcineurin/NFAT (nuclear factor of activated T cells) signaling pathway – all core features required for lymphatic valve development [103]. Studies in zebrafish have revealed that binding of the ligand ephrin B2 (EFNB2) to its receptor Ephrin type-B receptor 4 (EPHB4) suppresses ERK signaling through activation of RAS p21 protein activator 1 (RASA1). This signaling axis is critical for the differentiation and development of lymphatic valve cells. Pharmacological inhibition of ERK signaling (e.g., with selumetinib) promotes normal valve development, whereas excessive ERK activation results in valve hyperplasia or hypoplasia [101]. Transcription factors including GATA2, FOXC2, and PROX1 are upregulated in valve‑forming cells and cooperatively regulate valve development; these factors interact with the EFNB2–EPHB4–RASA1 axis to ensure proper valve morphogenesis [101, 104–106]. In addition, EFNB2–EPHB4 signaling influences cytoskeletal contractility and junctional integrity in lymphatic endothelial cells, maintaining the structural stability of mature valves [101].

**Fig. 5:**
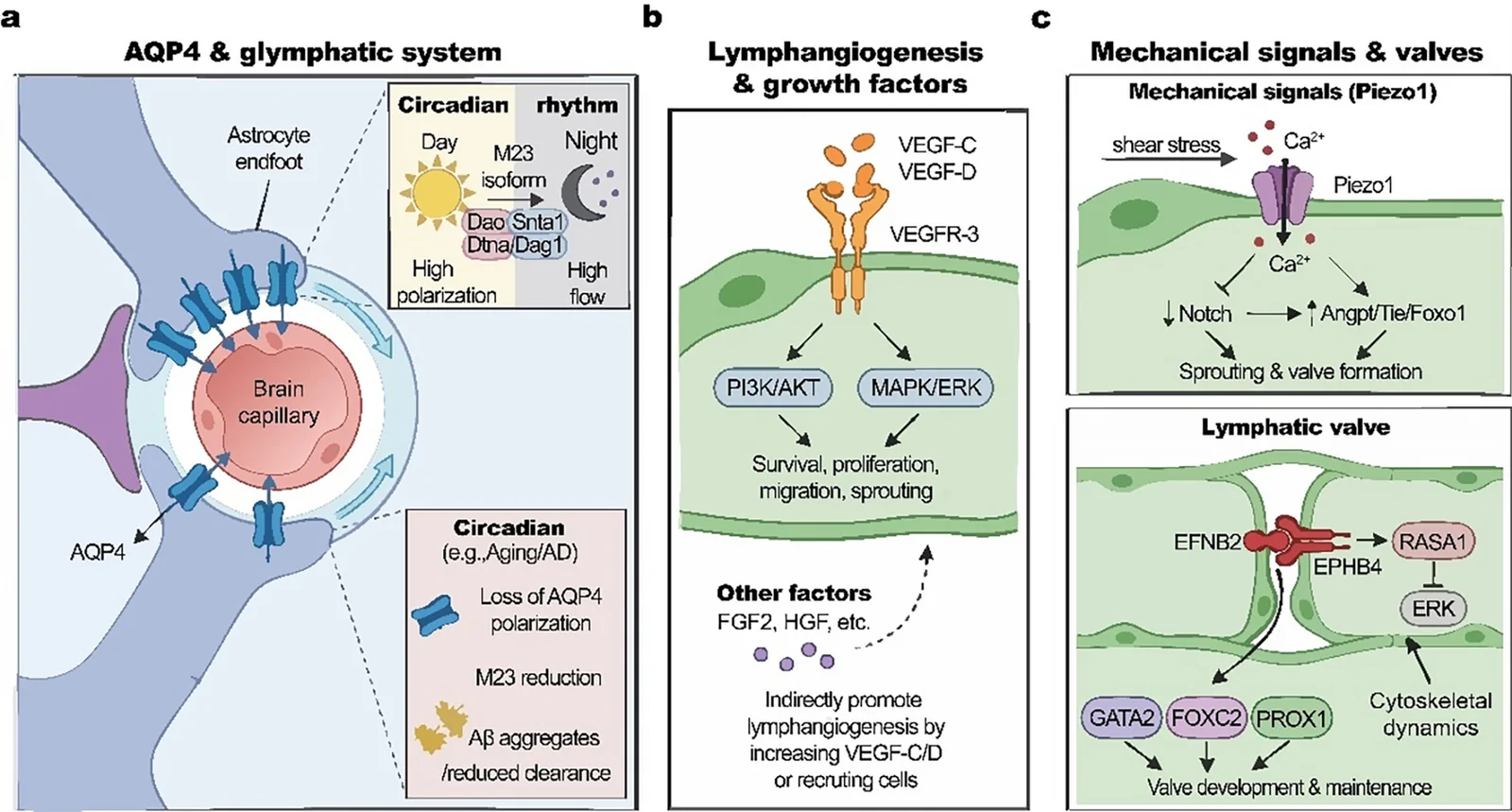
The mechanism related to the lymphatic system. **a** AQP4 and the glymphatic system, involving astrocyte endfeet, brain capillaries, AQP4 distribution, and the influence of circadian rhythm on it. It also points out the changes under pathological conditions such as aging or AD. **b** Mechanism of lymphangiogenesis and growth factors, including VEGF-C and VEGF-D and their downstream signaling pathways, as well as other factors such as FGF2 and HGF that indirectly promote lymphangiogenesis. **c** Mechanisms related to mechanical signals and valves, covering the role of signal transduction triggered by mechanical signals (Piezo1) in lymphatic vessel sprouting and valve formation, as well as the molecular and cellular processes involved in the development and maintenance of lymphatic valves

Collectively, these findings indicate that lymphatic valve development relies on a precisely orchestrated molecular network, in which mechanical signals (transduced by Piezo1 and flow-mediated pathways) act upstream of a core transcriptional and signaling cascade centered on EFNB2–EPHB4–RASA1-mediated ERK suppression. Transcription factors and cytoskeletal regulators act in concert to establish and maintain functional valve architecture, providing an important foundation for understanding lymphedema pathogenesis and developing targeted therapeutics to restore valve function (Fig. 5).

## Physiological functions and immune surveillance

### CSF circulation and clearance

#### Basic production and classical circulation pathway

CSF is primarily produced by the choroid plexus located within the lateral, third, and fourth ventricles, with approximately 500 mL secreted daily in healthy adults [107]. Its composition is tightly regulated by the blood–CSF barrier and is characterized by high concentrations of ions such as sodium and chloride, along with low levels of proteins, whereas macromolecules such as immunoglobulins are present at markedly lower concentrations than in plasma [107, 108]. This biochemical profile makes CSF an ideal medium for the clearance of metabolic waste from brain tissue. Freshly produced CSF flows through the ventricles and subarachnoid space, propelled by a combination of arterial pulsation and respiratory movement [108]. Specifically, CSF travels from the lateral ventricles to the third ventricle, passes through the cerebral aqueduct into the fourth ventricle, and then enters the subarachnoid space via the foramina of Luschka and Magendie, distributing widely around the brain and spinal cord [108]. A portion of the CSF subsequently enters the brain parenchyma through the PVS formed by astrocytic endfeet, where it mixes with ISF, constituting the “influx gateway” of the glymphatic system [109].

#### Physiological regulation of CSF flow and clearance

The flow and exchange of CSF are governed by a set of finely coordinated physiological mechanisms. Arterial pulsation serves as the primary driving force, with cardiac systole generating waves of vascular expansion and contraction that propel CSF through PVS into the deeper regions of the brain parenchyma [110]. Respiratory rhythms and slow vasomotor oscillations further contribute to CSF influx along periarterial pathways [109]. Notably, this clearance process is substantially enhanced during sleep and is essential for maintaining brain homeostasis [111, 112]. Particularly during non-rapid eye movement (NREM) sleep, the interstitial space expands by approximately 60% [113], which in turn drives the increased CSF influx into the brain parenchyma [112]. Subsequent studies revealed that the polarized expression of astrocytic AQP4 is regulated by circadian rhythms, becoming more pronounced during sleep and thereby facilitating glymphatic waste removal; this mechanism is robustly demonstrated in mice [114]. In recent years, human studies have established direct links between electroencephalogram (EEG) activity and CSF dynamics during NREM sleep [115, 116]. Norepinephrine-mediated slow vasomotion has been identified as a primary mechanical driver of glymphatic clearance during NREM sleep, with tight synchronization among vascular pulsations, CSF flow, and EEG activity [117]. During NREM sleep, major neuromodulators exhibit synchronized fluctuations that are phase-coupled to CSF flow [118].

Although the primary clearance routes rely on periarterial CSF influx and perivenous efflux, contraction of astrocytic endfeet may facilitate the direct removal of small metabolic solutes by modulating perivascular fluid dynamics, for example, through potassium-dependent vasodilation and vasoconstriction [119, 120]. During cerebral edema, AQP4-dependent calcium (Ca^2^⁺) signaling in astrocytic endfeet is rapidly activated, and this pathological signaling may cause endfoot swelling, disrupt capillary hemodynamics, and consequently impair perivenous clearance efficiency [121]. In the *Aqp4*-knockout mice, clearance of both small and large solutes (including 500 kDa Dex) is markedly reduced, underscoring the essential role of AQP4 in endfoot-mediated waste removal [64]. Astrocytic endfeet form a highly organized perivascular barrier composed of scaffold proteins that anchor ion channels, transporters, and enzymes. The inter-endfoot clefts, approximately 20 nm in width, function as a secondary extracellular barrier that may regulate the diffusion efficiency of small metabolites such as lactate by modulating the extracellular volume fraction [122, 123]. Under elevated intracranial pressure, altered endfoot contractile activity can initiate Ca^2^⁺ signaling, such as transient receptor potential vanilloid 4 (TRPV4)-mediated Ca^2^⁺ influx, which triggers the release of vasoactive factors like prostaglandins, thereby modulating local blood flow and clearance capacity [124]. Moreover, loss of endfoot coverage or mislocalization of polarity proteins, such as AQP4, exacerbates BBB disruption and leads to pronounced clearance deficits [125].

#### Dural MLVs: a key extracranial CSF drainage pathway

The relative contribution of dural MLVs versus other CSF outflow routes (e.g., the nasopharyngeal/cribriform pathway) to the overall CSF clearance remains an actively debated question in the field. Nevertheless, convergent evidence from murine models, non-human primates, and human postmortem dural tissue mapping has established dural MLVs as an important extracranial drainage route for CSF and its metabolic waste [27, 42]. Crucially, functional assessment using DCE-MRI in mice demonstrates that basal MLVs, located along the petrosquamosal and sigmoid sinuses, exhibit significantly higher CSF uptake and drainage efficiency than dorsal MLVs [27]. Human ex vivo dural tissue analysis further confirms denser LYVE-1⁺/PROX1⁺ vessel networks in basal versus dorsal dura [42]. These structural and functional data collectively support basal MLVs as a high-capacity conduit for CSF and macromolecular drainage under physiological homeostasis, although whether they represent the quantitatively dominant pathway in humans requires further direct validation. In contrast, the ACE provides a bidirectional exchange interface between the subarachnoid space and the dura mater, enabling rapid transfer of water, solutes, and even cells between the CSF and the dural compartment—though its quantitative contribution to global CSF outflow remains undefined in humans due to technical limitations of in vivo measurement [29].

#### Anatomically validated auxiliary drainage pathways: NLVs and other routes

NLVs represent an anatomically validated auxiliary pathway for CSF drainage, particularly well documented in rodents [12, 126]. Driven by intracranial pressure, the pressure within the subarachnoid space exceeds that of the nasal interstitial compartment, creating a pressure gradient that allows CSF to flow through perineural spaces surrounding the olfactory nerves at the cribriform plate and enter the NLVs, forming the brain–nasal drainage route [127]. This pathway bypasses the BBB and enables the exchange of metabolic waste, proteins, and certain pharmacological agents contained within CSF [127]. A portion of CSF can also be directly absorbed by the submucosal lymphatic microvessels opening near the cribriform plate, which subsequently drains into the deep cervical lymphatic vessels and ultimately reach the dCLNs [126]. Further studies have shown that the patency of the NLVs directly influences CSF flow velocity: larger-caliber lymphatic vessels tend to support faster lymphatic transport, whereas narrowed or collapsed vessels significantly reduce the drainage efficiency [128].

While the anatomical existence of both dural MLVs and nasopharyngeal/cribriform lymphatic pathways is now well established across multiple species, the field remains actively divided regarding their relative contribution to overall CSF outflow, particularly in humans [1]. On the one hand, studies using fluorescent CSF tracers in Prox1-GFP lymphatic reporter mice have identified the NPLP as a “major hub” for CSF outflow to dCLNs, highlighting its central role in the brain‒nasal drainage axis [12]. This pathway is anatomically plausible across species, and its age‑related regression suggests functional relevance in aging, though pharmacological activation of deep cervical lymphatic transport can partially compensate [12]. On the other hand, functional assessment using DCE‑MRI in mice demonstrated that the basal MLVs exhibit significantly higher CSF uptake and drainage efficiency than the dorsal MLVs, and human ex vivo dural tissue analysis confirmed denser lymphatic vessel networks in basal versus dorsal dura [37, 42]. These structural and functional data support basal MLVs as a high‑capacity conduit for CSF macromolecular clearance under physiological homeostasis.

Importantly, the interpretation of relative contributions differs markedly depending on species and methodological approach. In mice, tracer studies suggest that a substantial portion of CSF outflow occurs via lymphatic vessels along the sheaths of cranial nerves and the cribriform plate, indicating a significant, but not exclusive, contribution of the nasal route [52]. In contrast, human evidence remains limited to indirect MRI metrics (e.g., CSF‑STREAM signal dynamics near the cribriform plate) and postmortem lymphatic mapping, neither of which allows volumetric quantification of CSF flux through individual routes [20]. Thus, while dural MLVs are widely regarded as an important extracranial drainage route, and the nasopharyngeal plexus has been characterized as a major hub, the quantitative hierarchy between these pathways—particularly in humans—remains unresolved and likely varies with species, age, and pathological context. Future studies using advanced human imaging techniques are critically needed to clarify the functional prioritization of these drainage routes under physiological and disease conditions.

Notably, both superficial CLNs and dCLNs serve as relevant downstream drainage stations for cranial and nasal lymphatic CSF clearance—with their relative contribution depending on anatomical route, tracer location, species, and experimental conditions. Tracer-mapping studies in Prox1-GFP lymphatic reporter mice demonstrate that CSF transported through nasal-associated lymphatic pathways can drain to both superficial CLNs (particularly via nasal, periorbital, and hard-palate routes) and dCLNs (via the nasopharyngeal lymphatic plexus and downstream cervical lymphatics) [12, 129]. Human evidence for this dual-drainage architecture remains inferential, relying on postmortem lymphatic mapping and indirect imaging, but the existence of both superficial and dCLN drainage is now consistently observed across multiple rodent models and supported by comparative anatomy in non-human primates [12, 129].

The lymphatic vessels within the optic nerve sheath form a secondary drainage route for CSF, commonly referred to as the brain–eye pathway [49]. Recent studies have highlighted the important role of the optic nerve and its surrounding tissues in CSF circulation [18, 50]. By intraventricular injection of dextran tracers, Mathieu et al. [51] demonstrated that CSF enters the optic nerve parenchyma via PVS surrounding penetrating microvessels, providing evidence of a glymphatic pathway within the optic nerve. Aspelund et al. [10] and Ma et al. [52] further used CSF-tracking methodologies to show that MLVs cluster around the optic nerve and drain CSF extracranially; CSF tracers injected into the subarachnoid space can exit through the optic nerve and reach periocular tissues and CLNs [130]. Additional work using contrast agents of varying molecular weights and immunofluorescence labeling has demonstrated that tracers smaller than 70 kDa can traverse lymphatic pathways into the optic nerve parenchyma [51]. These findings indicate that lymphatic-mediated CSF transport within the optic nerve is both functional and molecular size-dependent. Although earlier studies reported only sparse lymphatic capillaries on the optic nerve in humans and non-human primates [131, 132], more recent evidence supports the presence of a lymphatic network within the optic nerve sheath, located along the perineural membranes rather than within the nerve itself [49]. Moreover, optic nerve sheath lymphatics have been shown to effectively drain intravitreal substances, such as fluorescent tracers and antigens, into the dCLNs [49].

The trigeminal nerve, acting as a conduit between the brainstem and cranial nerve parenchyma, participates in CSF–ISF exchange and provides a critical pathway for CSF drainage [133]. Tracer studies in animal models have shown that the trigeminal route coexists with the cribriform/nasal drainage pathway to the cervical lymph nodes, together contributing to the elimination of waste products from the brain [133, 134]. In migraine animal models, CSF has been observed to enter the trigeminal ganglion directly, where it activates trigeminal neurons through nonsynaptic signaling mechanisms [133]. This finding suggests that dynamic changes in CSF flow may influence pain transmission via the trigeminal system, offering new insights into the pathophysiology of migraine.

Spinal CSF circulation represents an integral component of the glymphatic system. Through perineural spaces surrounding the spinal nerves, CSF exchanges with CNS ISF and contributes to the removal of metabolic waste products [129]. This process is thought to be driven in part by pulsatile forces generated by blood vessels adjacent to spinal nerves. Anatomical evidence suggests that structures surrounding the spinal nerves may participate in CSF clearance through lymphatic vessels [135]. CSF may drain either via lymphatic pathways around spinal nerves or directly into the peripheral lymphatic system [135]. Recent studies have further revealed that, beyond the classical foramina of Magendie and Luschka, CSF can exit the spinal compartment through periaxonal spaces around spinal nerves, forming clearance routes that connect the spinal cord to peripheral organs such as the liver and pancreas [136]. Clinical studies in patients with spontaneous intracranial hypotension show that spinal CSF leaks accelerate CSF clearance into the bloodstream while reducing glymphatic inflow [137]. These findings suggest that CSF leakage in the spinal nerve region can alter pressure gradients and thereby influence the global clearance efficiency. Additional evidence indicates that CSF flow can cross the attachment points of spinal nerve roots, enter the fibrous layers of peripheral nerves, and ultimately reach the axoplasm of distal nerve fibers [138]. Although research to date has focused primarily on cranial lymphatic pathways, spinal CSF efflux routes including those along spinal nerves are gaining increasing recognition. Further exploring the contribution of spinal nerve–associated pathways to CSF clearance will be critical for achieving a comprehensive understanding of the glymphatic system and its roles in both healthy and pathological conditions.

### Immune surveillance

The immune surveillance of the CNS is not a monolithic process but a hierarchically organized, anatomically segregated, and temporally regulated defense architecture, wherein distinct cellular sentinels operate within non-overlapping yet functionally coupled compartments—each with unique ontogeny, activation thresholds, and pathological consequences.

#### Parenchymal immune surveillance: intrinsic sentinel function of microglia

Microglia serve as the CNS’s intrinsic immune sentinels, constantly surveying the parenchyma via dynamic process motility [139, 140]. Their immunosurveillance capacity is intrinsically coupled to metabolic reprogramming: upon activation, they upregulate glycolytic enzymes (e.g., hexokinase 2) to promote migration, phagocytosis, and cytokine production [141]. While essential for homeostasis, such as clearing Aβ aggregates and neuronal debris via scavenger receptors (SR-A1, CD36) and initiating adaptive immunity through major histocompatibility complex II (MHC-II) upregulation and IL-1β/TNF-α release [142], their functional plasticity renders them double-edged. Physiologically, microglia refine neural circuits via complement-dependent synaptic pruning, a process vital for developmental wiring and adult plasticity [143, 144]. However, in AD models, chronic exposure to Aβ and tau triggers sustained hyperactivation of complement pathway, leading to excessive, non-selective synapse elimination, particularly in early disease stages preceding overt neuronal loss [145, 146]. Crucially, genetic or pharmacological inhibition of C1q, C3, or CR3 rescues synaptic density and cognitive deficits in murine AD models, directly implicating dysregulated microglial phagocytosis as a causal mechanism of circuit failure [147]. This dual role underscores that microglial pathology in neurodegeneration is not defined by simple “activation” but by a loss of functional specificity: the same molecular machinery (complement, scavenger receptors) that maintains health becomes co-opted to drive degeneration when chronically stimulated or inadequately resolved.

#### Meningeal immune surveillance: anatomically organized border compartments

Beyond the parenchyma, the meninges constitute a sophisticated immunological interface, housing diverse innate and adaptive immune cells including monocytes, neutrophils, macrophages, T cells, and B cells, which collectively surveil the CNS borders [148, 149]. Critically, this cellular diversity is spatially organized, not diffusely distributed. Recent work has identified “peri-sinus” immune niches specifically localized around dural venous sinuses, comprising highly concentrated clusters of CD4⁺ and CD8⁺ T cells, regulatory T cells (Tregs), MHC-II⁺ macrophages, and conventional dendritic cells (cDC1/cDC2) [150]. These niches are not static reservoirs but functional antigen-processing units: cDCs efficiently capture CSF-borne antigens, present them to T cells, and license local B-cell responses, thereby enabling rapid, regionally tailored immune reactions without requiring peripheral lymph node recirculation [150]. This organization explains how the meninges can mount robust defenses against CNS infection while minimizing systemic inflammation. Supporting this, parabiosis experiments revealed a locally maintained, self-renewing pool of myeloid progenitors within the meninges, continuously replenished by hematopoietic stem/progenitor cells from adjacent skull and vertebral bone marrow via specialized vascular channels (40–150 μm diameter) [148]. In stroke models, neutrophils are preferentially recruited directly from skull marrow—not peripheral blood—to injury sites, highlighting the meningeal compartment’s role as an on-site, rapidly deployable immune reserve [151]. Furthermore, skull bone marrow is not a passive drainage site but an active immune‑sampling interface where resident B cells capture brain‑derived antigens and up‑regulate the tolerogenic checkpoint molecule PD‑L1, likely helping to maintain CNS immune tolerance [60].

Dural-associated lymphoid tissue (DALT) represents a further layer of meningeal immune specialization. It is an anatomically discrete, inducible lymphoid structure primarily located near the rostral confluence of nasal sinuses and connected to skull marrow vasculature [152]. This structure receives vascular perfusion via the superior sagittal sinus and bridging veins, and integrates into a complex immune network that provides robust protection to the CNS [152]. Unlike transient inflammatory infiltrates, DALT develops postnatally in mice and undergoes age-dependent functional maturation, progressively enhancing its capacity for germinal center formation, B-cell activation, and plasma cell differentiation upon antigenic challenge [152]. The enrichment of B cells and the ability to expand dramatically during immune challenge position DALT not merely as a surveillance site but as a primary CNS barrier organ. By generating antigen-specific antibodies (e.g., IgA) locally, DALT can neutralize pathogens before they breach deeper barriers, providing a critical first line of humoral defense. This functional maturation parallels the age-related increase in AD risk, suggesting that DALT immunosenescence, rather than mere accumulation of pathology, may contribute to the declining CNS immune vigilance in aging.

ACE, which is a structural discontinuity in the arachnoid layer surrounding bridging veins, is a well-validated interface enabling bidirectional exchange of CSF, solutes, and immune cells between the subarachnoid space and the dura mater [29]. ACE provides a mechanistic basis for how meningeal immune cells access CSF-derived antigens without compromising the overall barrier integrity, functioning as a regulated “gate” rather than a passive leak.

#### Mucosal immune surveillance: the brain–nasal axis mediated by nasal-associated lymphoid tissue (NALT)

NALT serves as the first immunological checkpoint for inhaled antigens [153]. Its strategic location, rich B-cell content, and direct lymphatic connectivity to dCLNs, make it a dedicated brain–nasal immunosurveillance axis [154, 155]. Upon viral invasion, human NALT rapidly initiates adaptive responses, secreting IgA to neutralize pathogens directly in the nasal mucosa [156]. Critically, NALT does not act in isolation: it conveys antigenic information to the dCLNs via lymphatic drainage, thereby indirectly shaping the CNS immune surveillance [155, 156]. Nanoparticles delivered intranasally bypass the BBB and enter the brain parenchyma via olfactory/trigeminal nerves [157, 158], and NALT likely modulates this process through local antigen presentation or cytokine conditioning of draining immune cells. Furthermore, NALT interfaces with the olfactory mucosa, where resident type 2 innate lymphoid cells and T cells maintain a balance between neuronal support and inflammatory control, protecting both upper airways and the brain from pathogenic invasion [155, 159]. Thus, NALT is not merely a mucosal defender but a key integrator linking environmental exposure, peripheral immunity, and CNS homeostasis.

#### Neural route immune surveillance: trigeminal and spinal nerves as immunological highways

Cranial and spinal nerves extend the immune surveillance network beyond traditional boundaries. The trigeminal nerve, uniquely equipped with a transition zone (TZ) where its CSF-exposed segment interfaces with peripheral tissues, serves as a privileged conduit for immune cell infiltration [160]. In autoimmune encephalomyelitis models, CCR2⁺ monocytes accumulate bilaterally at the TZ, mirroring clinical cranial neuropathies and identifying the trigeminal nerve as a critical node in autoimmune neuroinflammation [160]. Similarly, in trigeminal neuralgia, CD4⁺/CD8⁺ T cells and CD68⁺ macrophages infiltrate nerve compression sites, with CD68⁺ cells exhibiting a distinct phenotype not overlapping with F4/80⁺ macrophages—suggesting nerve-specific macrophage programming [161]. Spinal nerves also participate: CSF exchanges with ISF via perineural spaces, and recent evidence shows CSF can exit the spinal compartment through periaxonal spaces to reach peripheral organs like the liver and pancreas [136]. Clinically, spinal CSF leaks in intracranial hypotension accelerate CSF clearance into the bloodstream while reducing glymphatic inflow, demonstrating how peripheral nerve–associated efflux routes directly modulate global CNS clearance efficiency [137]. This positions peripheral nerves not as passive cables but as active immunological highways, facilitating bidirectional communication that underlies neuropathic pain, autoimmunity, and systemic metabolic crosstalk.

In summary, immune surveillance of the CNS is a hierarchically organized, evolutionarily conserved system spanning parenchymal microglia, structured meningeal niches (peri-sinus hubs, DALT), mucosal interfaces NALT, and neural conduits (trigeminal/spinal nerves). Its dysfunction in neurodegeneration is characterized not by global immunosuppression but by compartment-specific maladaptation: microglial loss of synaptic specificity, meningeal niche dysregulation, DALT immunosenescence, NALT–CNS axis breakdown, and aberrant nerve-immune crosstalk. Therapeutic strategies must therefore move beyond broad anti-inflammatory strategy to targeted immunomodulation within the precise anatomical and immunological context defined by current evidence, including restoring microglial phagocytic fidelity, enhancing meningeal antigen presentation, rejuvenating DALT function, and modulating the NALT–dCLN signaling.

## Aging and neurodegenerative disorders

### Aging

Aging is the single largest modifiable risk factor for most neurodegenerative diseases. With advancing age, both the glymphatic system and the MLVs undergo coordinated structural and functional deterioration, leading to markedly reduced efficiency in metabolic waste removal [162]. Critically, this degenerative process is not uniform across the meningeal immune compartment. Distinct cell populations, anatomical niches, and efflux routes exhibit differential sensitivity to aging, resulting in compartment-specific dysfunction that synergistically promotes pathological protein accumulation and cognitive decline.

#### AQP4 polarity loss: regional specificity and functional dissociation from total protein abundance

In aged C57BL/6 J mice (18–20 months), although the overall expression level of AQP4 does not differ markedly from that in young animals, the perivascular AQP4 polarity around penetrating arterioles is profoundly disrupted. This loss of polarization is most pronounced in the lateral and ventral cortex, hippocampus, and striatum, representing a major driver of glymphatic dysfunction, albeit not an absolute requirement for age‑related impairment of glymphatic transport [163]. In humans, loss of the perivascular AQP4 polarity correlates with increased Aβ burden and higher Braak stage, independent of age, whereas the polarity is largely preserved in cognitively intact oldest‑old individuals (> 85 years) [164]. Notably, multiple postmortem studies have reported that the total AQP4 expression increases with age in cognitively normal older adults, with particularly pronounced elevations in the frontal cortex and choroid plexus [164–166]. This observation suggests that maintaining the correct spatial localization of AQP4 may be a key protective mechanism against neurodegenerative processes. In aging and CAA, the polarized distribution of AQP4 is markedly disrupted, characterized by redistribution from astrocytic endfeet to the soma and proximal processes, thereby impairing perivascular clearance [166, 167]. Additional aging‑related factors, including reduced arterial pulsatility and slowed CSF transport, further contribute to glymphatic impairment in the elderly [163, 168]. While most studies have not specifically addressed sex differences, one postmortem analysis of 8 individuals (3 males, 5 females, aged 74–91 years) detected AQP4 expression in the choroid plexus across all samples, suggesting that these age-related changes may occur independently of biological sex [169].

#### MLVs: regional heterogeneity in aging vulnerability

In aged C57BL/6 J mice (20–24 months), MLVs, particularly those located in the basal region, exhibit reduced LYVE-1-positive coverage and diminished drainage of the tracer Alexa Fluor 647-Ovalbumin to the dCLNs, changes that correlate with cognitive impairment [170]. Basal MLVs, which serve as a major “hotspot” for macromolecular clearance from CSF, show age-dependent deterioration in both structural integrity and drainage efficiency [27, 170]. Notably, this degeneration appears to be region- and organ-specific: while the basal MLVs display marked age-dependent rarefaction and functional decline, capillary lymphatics in peripheral tissues such as the skin and trachea do not show severe structural remodeling in aged mice, suggesting that the CNS-associated lymphatic beds are particularly vulnerable to aging-related insults [170–172]. Lymphatic valves are also markedly affected by aging. Aged mice exhibit abnormal collagen deposition and a reduced number of valves in basal MLVs compared to young mice [173]. Expression of the transcription factors Prox1 and Foxc2, both critical for lymphatic valve maintenance, is significantly decreased in aged mice. In particular, Prox1 expression declines progressively with age [103].

Consistent with these structural changes, high-resolution anatomical and functional imaging in Prox1-GFP reporter mice demonstrated a 40%–60% reduction in the total CSF lymphatic outflow in aged mice (18 months) compared with young mice (2 months) following intraventricular injection of near-infrared tracers [52]. Moreover, the basal MLVs are consistently more vulnerable to aging than the dorsal MLVs, with a disproportionately greater decline in CSF drainage efficiency [174]. These findings confirm that lymphatic vessels in different anatomical regions exhibit distinct sensitivities to aging, potentially driven by local mechanical forces (e.g., CSF flow pressure) or age-related alterations in the regional immune microenvironment.

#### Age-related remodeling of the meningeal T-cell and B-cell compartment: from homeostasis to chronic inflammation

Senescence drives a global shift of meningeal T cells from a naïve to an effector-memory phenotype, resulting in accumulation of Th2 cells, Tregs, and a specialized CD153⁺CD4⁺ T-cell subset, which may initially exert compensatory protective roles by sustaining the brain homeostasis [175]. Single-cell RNA sequencing further revealed the characteristic transcriptional profiles of CD8⁺ CD69⁺ CD103⁺ tissue-resident memory (TRM) cells in the aging meninges [162]. These cells have a direct spatial association with lymphatic endothelial cells, providing a structural basis for T cells to affect lymphatic vessel function through paracrine signaling [162]. Concurrently, aging leads to progressive loss of MLV structural integrity and reduced CCR7 expression in meningeal T cells, impairing their migratory capacity out of the meninges and further exacerbating glymphatic dysfunction [176]. Elevated interferon-gamma (IFN-γ) levels in the aged meninges, which are associated with the increase in total T cells, may directly reduce the basal MLV lymphatic flow, although the exact molecular mechanism linking IFN-γ to endothelial dysfunction remains to be fully characterized [162]. Age-dependent alterations in the C-X-C chemokine receptor type 6 (CXCR6)/CCR6 expression profile of γδ T cells further alter their migratory behavior, potentially increasing their propensity to infiltrate the parenchyma and contribute to aging-related brain injury [177].

In addition to T-cell alterations, aging exerts profound effects on meningeal B cells. Studies indicate that while a fraction of B cells in aged mouse meninges originates from the blood, the majority is supplied by bone marrow-derived cells that migrate through specialized vascular channels connecting the calvarial marrow with the meninges [178]. This calvaria–meninges conduit may provide the CNS with a continuous influx of B cells chronically exposed to CNS antigens [178]. Furthermore, aging is associated with evidence of B-cell developmental trajectories along the dural venous sinuses, suggesting that the meninges may host a bone marrow-like microenvironment capable of supporting local B-cell maturation [179]. Aging induces marked transcriptomic reprogramming in meningeal B cells, characterized by the activation of both innate and adaptive immune pathways. For example, genes associated with antibody production and interferon signaling are significantly upregulated in aged meninges [180]. Loss of specific B-cell subsets during aging also has functional consequences. As a key subset of the innate immune system, the number and the function of B-1a cells decrease with aging, leading to reduced secretion of natural antibodies and decreased immune surveillance ability. This makes the elderly more susceptible to pathogens, and the difference in mortality is more significant in male individuals [181]. Notably, B220^low^ B-cell populations that accumulate in young mice during disease remission are absent in aged mice, suggesting impaired immunoregulatory capacity with age [182]. Reduced CCR7 expression in aging may further disrupt T- and B-cell migration, thereby worsening the cognitive decline and Aβ pathology [176]. In addition, the accumulation of meningeal ectopic lymphoid structures (ELS) correlates with age, brain pathology, and sex, and may promote the expansion of autoreactive B-cell clones through germinal center–like reactions [183].

#### Innate meningeal immune niche remodeling in aging

In aged C57BL/6 mice (17–20 months) NK-cell subsets is significantly reduced, concomitant with decreased expression of miR-181a-5p, which leads to defects in the development and effector function of NK cells [184]. Restoring IFN-γ signaling in NK cells or repairing age-related meningeal lymphatic dysfunction, for instance, through CCBE1 (collagen and calcium-binding EGF Domain 1)-mediated enhancement of lymphatic drainage, has been shown to mitigate the aging-associated neurodegeneration and functional decline [162, 185, 186]. Meningeal myeloid cells may actively promote the formation and maintenance of ELS through antigen presentation or secretion of chemokines such as C-X-C motif chemokine ligand CXCL13 [187].

**Fig. 6:**
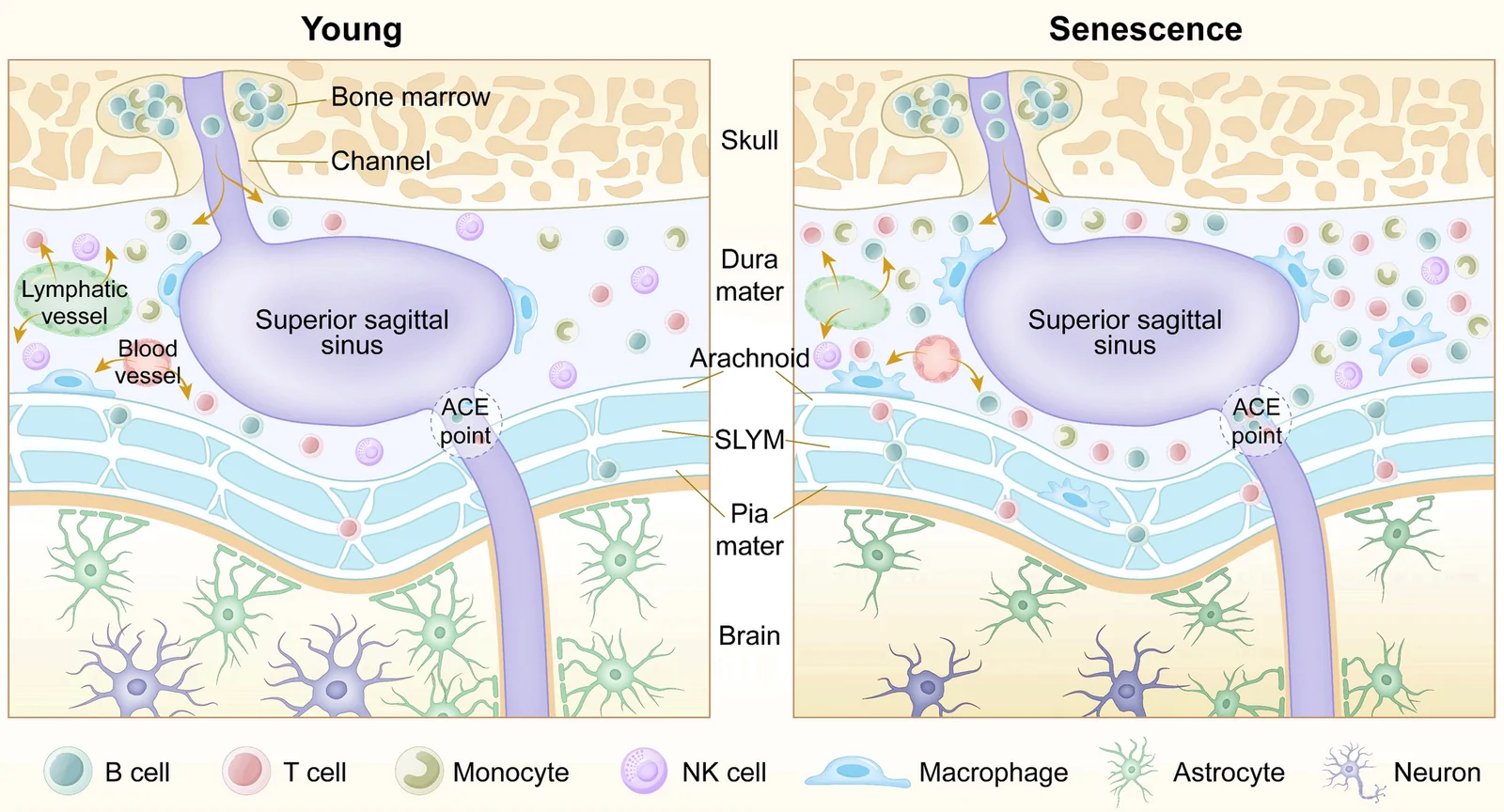
Comparisons of the meningeal lymphatic system and the distribution of related immune cells in young and aging states

Fate-mapping, single-cell transcriptomics, and cell-specific genetic perturbation studies have demonstrated that meningeal macrophages and parenchymal microglia share a common embryonic progenitor, whereas perivascular macrophages arise postnatally from migration and differentiation of meningeal macrophages [188]. This differentiation trajectory requires integrin-dependent mechanisms and involves reciprocal interactions with arterial smooth muscle cells [188]. During aging, meningeal fibroblasts exhibit reduced expression of integrin β1 alongside increased expression of the collagen receptor discoidin domain receptor 2, potentially altering the extracellular matrix adhesion properties and thereby reshaping macrophage localization and functional polarization [189]. Furthermore, aging drives meningeal macrophages toward a senescence-associated secretory phenotype characterized by increased migrasome release and secretion of pro-inflammatory mediators, which modulate the phenotype and function of neighboring stromal and immune cells [190]. Age-related thickening of the proposed SLYM is accompanied by immune-cell infiltration, particularly T-cell accumulation and macrophage phenotypic shifting, creating a persistent low-grade inflammatory milieu [37]. These changes further impair the barrier and fluid-exchange capacities of the meninges, contributing to progressive decline in both CNS immune surveillance and clearance functions (Fig. 6).

#### NLVs: a critical site of age-related drainage decline

In aged C57BL/6 J mice (aged 73 to 102 weeks), the numbers of lymphatic vessels in the nasal mucosa and hard palate are markedly reduced, resulting in diminished CSF efflux toward the CLNs [12, 42]. Concurrently, the NPLP undergoes pronounced age-related atrophy, whereas the dCLNs remain structurally unaffected [12]. Single-cell analyses further revealed that lymphatic endothelial cells within the aged NPLP exhibit robust upregulation of interferon signaling and inflammatory cytokine pathways [12]. These findings suggest that the nodal role of NLVs in CSF drainage progressively deteriorates with aging. Despite this decline, dCLNs retain a degree of compensatory capacity, partly enhancing the CSF outflow through adrenergic or nitric oxide-dependent mechanisms [12]. Aging-related structural impairment of NLVs is also reflected in their lymphatic valve architecture: aged mice show significantly fewer valves, directly compromising the collecting function of these vessels [12]. Comparative imaging studies demonstrate that the relative contribution of the nasal route to CSF clearance is substantially reduced in older animals compared with their younger counterparts [12]. Collectively, these findings highlight NLVs as a critical site of vulnerability in aging-associated CSF drainage deficits, providing an important mechanistic entry point for understanding age-related glymphatic and meningeal lymphatic dysfunction.

#### Aging-related sleep disruption and glymphatic decline

Aging is characterized by progressive reductions in NREM sleep duration and slow-wave activity (SWA), which directly impair glymphatic clearance [191]. Sleep fragmentation and diminished SWA can disrupt the convective exchange between CSF and ISF, as demonstrated in both animal models [192] and human studies [193]. Using simultaneous EEG‑fMRI, Fultz and colleagues identified coherent oscillations among electrophysiological, hemodynamic, and CSF dynamics during human NREM sleep: neural slow waves precede hemodynamic oscillations, which in turn are coupled to large (~ 0.05 Hz) CSF inflow waves in the fourth ventricle [194]. This coupling suggests that the sleep‑related neural activity directly influences CSF pulsatility, thereby facilitating waste clearance [194]. EEG studies have demonstrated that increased δ‑power during NREM sleep correlates with enhanced glymphatic solute exchange, whereas elevated β‑power during REM sleep correlates with reduced glymphatic function [195]. Mouse studies also revealed that glymphatic influx and clearance follow an endogenous circadian rhythm, peaking during the mid‑rest phase, while CSF drainage to cervical lymph nodes follows an opposite pattern [74]. Aging‑related circadian misalignment and sleep disruption exacerbate multiple glymphatic‑sensitive mechanisms, including perivascular AQP4 depolarization, reduced arterial pulsatility, and mitochondrial dysfunction, all of which are further aggravated by fragmented sleep architecture [70, 74]. Thus, in the aging brain, the loss of NREM slow‑wave sleep and the resulting glymphatic impairment are intrinsically linked, accelerating the accumulation of neurotoxic proteins such as Aβ and α‑syn and contributing to the pathogenesis of age‑associated neurodegenerative diseases.

#### Traumatic brain injury (TBI): an acute insult that recapitulates and accelerates aging‑related lymphatic dysfunction

TBI is a major risk factor for neurodegenerative diseases and one of the leading causes of long‑term disability and mortality worldwide [196]. Studies in closed‑head TBI mouse models have shown that AQP4 expression is markedly upregulated in reactive astrocytes, accompanied by loss of perivascular AQP4 polarization. These changes peak around 7 days post‑injury and do not differ significantly between mild and moderate TBI. In moderate TBI, BBB permeability, brain edema and intracranial pressure largely normalize within 7 days, suggesting that AQP4 alterations may represent a compensatory mechanism rather than a direct driver of edema formation [197].

Even mild TBI induces profound and persistent impairment of meningeal lymphatic drainage, beginning within hours and lasting for at least one month, with elevated intracranial pressure as a key contributor [198]. Ablation of MLVs significantly exacerbates TBI‑induced neuroinflammation and cognitive deficits, whereas intracisternal injection of an Adeno-associated virus (AAV) vector expressing VEGF‑C attenuates post‑TBI neuroinflammatory responses [198]. Mild TBI also triggers meningeal vascular injury, localized inflammation, and infiltration of peripheral T lymphocytes [199]. Single‑cell RNA sequencing revealed upregulation of interferon signaling pathways in meningeal macrophages after TBI, while inflammation‑related genes in fibroblasts and adaptive immune cells display a pattern of “controlled upregulation” [200]. Notably, macrophages derived from skull bone marrow can infiltrate the brain parenchyma through skull–meningeal channels after TBI, widely populating the injury site and exhibiting a pro‑inflammatory phenotype [201]. CD4⁺ T cells play dual roles: they can form immune clusters with B cells in the bone marrow, promoting Th17 differentiation and exacerbating inflammatory damage, whereas regulatory T cells facilitate tissue repair by inducing M2‑like macrophage polarization and secreting anti‑inflammatory factors such as IL‑10 and TGF‑β [202–205]. Sleep disorders frequently complicate TBI and further exacerbate glymphatic impairment, contributing to persistent neuroinflammation and long‑term cognitive deficits [206].

Collectively, these findings highlight that the TBI‑induced disruption of meningeal lymphatic drainage and skull‑to‑meninges immune cell trafficking recapitulates many of the aging‑related changes in lymphatic and immune function described above. This provides a mechanistic link between acute brain injury and the subsequent increased risk of chronic neurodegeneration.

### AD

AD is the most common age-related dementia, characterized by the accumulation of Aβ plaques and neurofibrillary tangles, accompanied by synaptic dysfunction and progressive cognitive decline [207, 208]. Recent advances have established that dysfunction of the central lymphatic network (encompassing both the glymphatic system and MLVs) is a key driver of AD pathogenesis [209–211].

#### AQP4 depolarization in AD pathology

The glymphatic system clears Aβ and other metabolic waste through the dynamic exchange between CSF and ISF. Dysfunction of this pathway results in impaired Aβ clearance that is tightly linked to AD progression [210]. Genetic ablation of AQP4 markedly exacerbates parenchymal Aβ deposition and memory deficits in APP/PS1 mice [212]. Interestingly, AQP4 deficiency simultaneously enhances microglial activation and Aβ phagocytic capacity in the cortex [213], suggesting a compensatory innate immune response in the setting of impaired perivascular clearance. Human postmortem data confirm that the total expression of AQP4 positively correlates with Aβ burden in the frontal cortex independent of age, a pattern not observed in normal aging [167], indicating that AQP4 dysregulation in AD reflects active pathology rather than an aging-related change. In humans, AQP4 exhibits disease- and region-specific expression patterns: this positive association with Aβ burden is not observed in late-onset AD, highlighting potential mechanistic heterogeneity across AD subtypes [167]. In severe CAA, AQP4 expression is markedly reduced in the gray matter, whereas in the white matter, regardless of CAA severity or white-matter hyperintensity, AQP4 levels tend to decline and its perivascular polarity becomes disrupted [166]. Loss of AQP4 polarity is strongly associated with failure of the IPAD pathway, a key route for vascular Aβ clearance [166]. Collectively, these findings underscore the multifaceted and context-dependent roles of AQP4 in AD pathology, and suggest that both its expression level and spatial polarization may represent important targets for mechanistic investigation and therapeutic intervention.

#### MLV dysfunction in AD progression

MLVs play an essential role in the clearance of Aβ and other toxic macromolecules from the CNS. Impairment of this pathway accelerates Aβ deposition within both the meninges and brain parenchyma, ultimately contributing to learning and memory deficits [214]. In the 5 × FAD mouse model, ablation or functional disruption of MLVs markedly aggravates Aβ accumulation, microglial proliferation, and neurovascular dysfunction [214]. In addition, age-related decline in MLV integrity is increasingly recognized as a major vulnerability factor for AD, and the *APOE* ε4 allele further compromises this system. *APOE4* carriers exhibit impairment of MLV-mediated Aβ clearance and elevated AD susceptibility [211, 215]. Mechanistically, in brain organoids derived from parental induced pluripotent stem cell (iPSC) lines, *APOE4*-carrying cells exhibit significant differences from non-*APOE4* cells in the expression of a range of genes associated with lymphatic endothelial cell function [215]. Specifically, the expression levels of genes such as *EFNB2*, *VEGFR3*, *FN1*, *ITGA9*, *LYVE1*, *PDPN*, *PECAM1*, *PTPRC*, and *GJA1* are markedly reduced in *APOE4*-carrying brain organoids [215]. These preclinical data from iPSC organoids suggest APOE4 may be associated with altered expression of the MLV-related genes, and whether this leads to in vivo premature MLV atrophy in human carriers remains to be verified. However, it should be noted that not all models show consistent MLV changes: in APdE9 and 5 × FAD mice, Aβ remains largely confined to perivascular regions without overt changes in MLV morphology or function [216]. This highlights potential model‑ and age‑dependent heterogeneity that warrants further investigation.

#### Remodeling of meningeal adaptive immune niches in AD

In AD, both adaptive and innate immune compartments within the meninges undergo profound remodeling. In the 3 × Tg-AD model, particularly in females, cells producing IL-17 (primarily γδ T cells) are markedly increased in both the meninges and brain parenchyma, suggesting that heightened pro-inflammatory responses may further exacerbate AD pathology [217]. Critically, intracerebroventricular neutralization of IL-17 can prevent short-term memory and synaptic plasticity deficits in the early stages of the disease, and delaying IL-17 neutralization can delay the occurrence of memory decline and amyloidosis-related splenomegaly [217]. In addition, mucosa-associated invariant T cells in the meninges express antioxidant molecules, potentially participating in the regulation of tau pathology by maintaining meningeal barrier integrity [218].

The meningeal B cell compartment is increasingly recognized as a key player in AD pathogenesis, exhibiting both protective and pathogenic functions in a context-dependent manner. Studies have shown that the meningeal B cells can suppress the production and accumulation of Aβ in AD. In young 5 × FAD transgenic mice, an increased number of B cells has been observed in meningeal tissues, and these B cells are capable of alleviating Aβ pathology and memory impairment [219]. Notably, meningeal B cells have been found to inhibit Aβ production in the prefrontal cortex, and intriguingly, they originate from peripheral lung tissue, where Aβ is drained from the brain via meningeal lymphatics and activates lung B cells through the TLR4/NF-κB signaling pathway. These lung memory B cells then migrate to the brain via the CXCL12–CXCR4 axis [220]. Recent mapping of dural ELS has revealed age‑, sex‑, and brain pathology‑specific patterns: 5 × FAD mice exhibit premature and robust ELS expansion, APP/PS1 mice show increased structural complexity alongside an age‑related decline in ELS, whereas tauopathy models display markedly reduced ELS formation [183]. Aging drives the progressive accumulation of aging‑associated B cells in the meninges, characterized by reduced B-cell receptor diversity and elevated somatic hypermutation, with substantial clonal overlap between the dural aging‑associated B cells and plasma cells, suggesting local activation and differentiation within the meningeal niche [221]. Importantly, a recent study demonstrated that Aβ deposition in the skull bone marrow promotes the proliferation and differentiation of aging‑associated B cells via IL‑6 signaling, and these aging‑associated B cells infiltrate the brain parenchyma to exacerbate Aβ pathology and cognitive deficits; blockade of IL‑6R suppresses B cell activity and delays disease progression in 5 × FAD mice [222]. Moreover, a systems immunology analysis of AD patients revealed a disturbance in B cell maturation that correlates with cognitive decline, independent of age and environmental exposure, further supporting the clinical relevance of B cell dysregulation in AD [223]. Collectively, these findings indicate that the meningeal B cell niche undergoes age‑ and pathology‑dependent remodeling, shifting from a protective to a potentially pathogenic state, and that the skull marrow‑derived B cells represent a novel therapeutic target for early AD intervention.

#### Dysfunction of border‑associated macrophages in AD

In the context of AD pathology, monocyte-derived macrophages may infiltrate the brain parenchyma via the meninges, particularly during later stages of the disease [224]. In aged AD mice, the numbers of meningeal and perivascular macrophages increase, potentially alleviating Aβ pathology through the recruitment of peripheral monocytes [225].

Border‑associated macrophages, encompassing perivascular macrophages and meningeal macrophages, have emerged as critical regulators of CNS immune surveillance and glymphatic function [226]. Single‑nucleus profiling of human leptomeninges has identified distinct subtypes of border‑associated macrophages with differential gene expression from microglia, and these cells express AD risk genes nominated by GWAS, suggesting that dysfunction of border‑associated macrophages may directly contribute to AD pathogenesis [227]. Studies have further demonstrated that dysfunction of parenchymal border macrophages (PBMs), including their decline during aging or in AD, is closely associated with impaired glymphatic clearance capacity [228]. In hemizygous B6SJL-Tg mice, depletion of PBMs leads to more severe Aβ plaque deposition. Analyses of human samples further confirmed that PBMs in AD patients exhibit markedly reduced expression of phagocytosis-related genes such as *CD163* [228]. In AppNL-F knock‑in mice, early glymphatic failure before extensive plaque deposition is accompanied by loss of PBMs, whereas mouse lines with preserved PBM function show better Aβ phagocytosis [227]. These findings suggest that glymphatic dysfunction in AD models is not solely determined by plaque burden, but may depend on Aβ species, disease stage, and PBM abundance or functional state.

The study further elucidated the mechanistic role of PBMs in glymphatic Aβ clearance: PBMs secrete mesencephalic astrocyte‑derived neurotrophic factor (MANF) to suppress astrocytic stress responses, thereby maintaining proper glymphatic function [229]. In the chronic phase of brain injury, downregulation of MANF in PBMs leads to exacerbated glymphatic dysfunction, sustained Aβ accumulation, and cognitive decline, and supplementation of MANF exerts therapeutic effects by improving glymphatic system function [229]. Additionally, lipid‑laden border‑associated macrophages have been observed during AD progression, and reducing the microglial lipid load enhances Aβ phagocytosis, suggesting that metabolic reprogramming of border macrophages may represent a novel therapeutic avenue [230]. However, PBMs or perivascular macrophages may also contribute to vascular pathology under certain conditions. In CAA model mice, Timp1⁺CD169⁺ and MMP9⁺CD169⁺ perivascular macrophages are associated with vascular amyloid deposition [231]. Aβ antibodies enriched at sites of CAA promote the recruitment and activation of perivascular macrophages, thereby increasing vascular permeability and susceptibility to microhemorrhage [231]. Conversely, a recent study demonstrated that meningeal macrophages with an M2‑like phenotype and high TGF‑β1 expression play a protective role in early AD by facilitating Aβ clearance, and secreting TGF‑β1 to enhance oligodendrocyte maturation and microglial phagocytosis, thereby counteracting social behavioral defects and myelin damage [232]. In line with the concept of suppressing pro‑inflammatory meningeal immunity, IL‑17 neutralization at early disease stages is sufficient to prevent short‑term memory and synaptic plasticity deficits, an effect that occurs independently of changes in Aβ or tau pathology [217]. Therefore, modulating the functional balance of border‑associated macrophages by promoting M2‑like protective phenotypes or inhibiting pro‑inflammatory pathways such as IL‑17, represents a promising therapeutic avenue for AD.

#### Synergistic clearance dysfunction of glymphatic and meningeal lymphatic systems for Aβ and tau

In addition to Aβ, tau clearance also relies on the cooperative action of the glymphatic and the meningeal lymphatic systems. Abnormal aggregation of tau protein is closely linked to impairments in glymphatic clearance function [233, 234]. Studies have shown that inhibition of glymphatic clearance markedly increases tau aggregation in the mouse brain and promotes interhemispheric propagation of tau within the hippocampus, while simultaneously exacerbating cognitive deficits and brain volume alterations [233, 234]. In addition, the clearance of extracellular tau depends on the glymphatic pathway, and dysfunction of this pathway may directly drive the progression of tau pathology [235]. In tau pathology models, the glymphatic system and MLVs function cooperatively to clear tau: the glymphatic system transports tau from the brain parenchyma into the CSF, after which MLVs facilitate its elimination from the CNS via drainage to the cervical lymph nodes [236]. Thus, combined dysfunction of the glymphatic and meningeal lymphatic systems may lead to reduced efficiency of tau clearance, becoming a key factor in neuronal degeneration and cognitive impairment in AD. For example, subarachnoid hemorrhage simultaneously damages both the glymphatic and the meningeal lymphatic systems, further impairing tau clearance capacity [237]. This suggests that activation or enhancement of the glymphatic/meningeal lymphatic systems may represent a promising therapeutic strategy for AD. In mouse models of tauopathy, DCE-MRI has revealed substantial impairment of glymphatic transport function, accompanied by reduced perivascular AQP4 polarization in the caudal cortex [25]. Further studies have shown that intraperitoneal administration of the AQP4 inhibitor TGN-020 decreases both the intracerebral contrast-agent signal and the CSF tau concentration in wild-type mice, whereas these effects are abolished in AQP4 knockout mice [25]. This firmly establishes the central role of AQP4 in CSF–ISF exchange and tau clearance.

Given that the glymphatic clearance is maximally active during sleep, disruptions in sleep architecture further compound tau and Aβ accumulation. Sleep architecture is profoundly altered in AD patients, with reductions in slow-wave activity and disrupted slow oscillation-spindle coupling that have been linked to glymphatic dysfunction [238, 239]. Accumulating evidence suggests that these sleep disturbances may contribute to the accumulation of Aβ and tau, potentially creating a vicious cycle that further impairs perivascular clearance and accelerates cognitive decline in AD [238, 240]. Thus, sleep disruption and glymphatic dysfunction mutually reinforce each other, exacerbating the failure of tau clearance in AD.

#### Involvement of peripheral lymphatic drainage pathways in AD

Neuropathological studies indicate that Aβ deposition occurs very early in the olfactory bulb, in parallel with prominent olfactory dysfunction in AD [241, 242]. The lymphatic systems of the nasal mucosa and olfactory bulb play essential roles in clearing toxic proteins such as Aβ [243]. A recent human positron emission tomography (PET) study using [1-^11^C]-butanol, a highly permeable tracer with no significant brain binding, demonstrated that the tracer kinetics in the brain (lateral orbital frontal cortex) and the nasal turbinates are positively correlated and reflect cerebral amyloid status, providing direct evidence that the nasal pathway serves as a potential CSF egress site in humans [244]. Aβ deposition has been detected in the olfactory epithelium of both AD patients and Tg2576 mice, and nasal Aβ levels correlate with brain Aβ burden, suggesting that nasal lymphatic clearance may be impaired in AD [245, 246]. Moreover, because the olfactory bulb is directly connected to the NLVs, dysfunction of NLVs may represent an early pathological trigger, impairing immune surveillance and the efficiency of metabolic waste clearance. Additionally, studies have shown that the “brain–eye” draining pathway is impaired in AD models, leading to Aβ accumulation within the optic nerve sheath and subretinal space, which induces ocular pathological changes and visual decline [18].

Abnormally phosphorylated tau protein may propagate through the brain–nose or brain–eye pathways. Studies have shown that the spread of tau pathology is closely associated with neuronal connectivity, potentially disseminating along functional connections (such as the olfactory pathway) or anatomical structures [247, 248]. In transgenic mouse models, early tau pathology emerges in the olfactory regions and progresses along olfactory pathways toward key AD-related areas such as the hippocampus [249]. Moreover, VEGF-C expression within the brain–eye lymphatic pathway has been implicated in regulating tau propagation [250]. Phosphorylated tau (p-tau) deposition has been detected in the retina, exhibiting a pattern parallel to tau pathology in the AD brain [250, 251]. However, experimental data indicate that retinal ganglion cells demonstrate partial resistance to tau propagation. Even after injection of AD-derived pathological tau, no tau aggregation is observed along the retina–superior colliculus pathway over a six-month period [251]. In the P301S and P301L tauopathy mouse models, retinal edema and blood–retina barrier disruption have been observed, whereas treatment with the tau oligomer monoclonal antibody ameliorates these pathological changes [252]. Collectively, these findings suggest that AD pathology exhibits broad, cross-system characteristics, affecting not only classical brain regions, but also peripheral neural and lymphatic pathways.

### PD

PD is characterized by the abnormal aggregation of α-synuclein (α-syn) into Lewy bodies and the progressive loss of dopaminergic neurons [253]. Studies have shown that in PD models, reduced efficiency of the glymphatic system forms a vicious cycle with the accumulation of α-syn, further exacerbating neuronal injury [254]. For example, in A53T transgenic mice, the polarized distribution of AQP4 in astrocytic endfeet within the substantia nigra is disrupted, leading to a marked reduction in CSF–ISF exchange efficiency [255]. In addition, ligation of the dCLNs aggravates α-syn accumulation, triggers microglial activation, and accelerates the loss of dopaminergic neurons [255]. Imaging studies further underscore the importance of the glymphatic system in PD. Diffusion tensor imaging analysis along the perivascular space (DTI-ALPS) has demonstrated a significant association between glymphatic dysfunction and the severity of motor symptoms in PD patients, although it reflects periventricular white-matter diffusion rather than a direct glymphatic flow measure [256]. Moreover, an experimental mouse study found a positive correlation between EEG δ-wave activity and glymphatic transport efficiency [257]. Notably, PD patients frequently exhibit reduced slow-wave activity, including diminished δ oscillations during sleep, indicating impaired glymphatic function [254].

Furthermore, dynamic contrast-enhanced MRI has shown that patients with idiopathic PD exhibit significantly reduced MLV blood flow along the superior sagittal sinus and sigmoid sinus, as well as markedly delayed perfusion of the dCLNs, compared with patients with atypical parkinsonian syndromes [258]. Such reductions in MLV drainage capacity may exacerbate the accumulation of toxic proteins such as α-syn, thereby promoting neuroinflammation and neurodegeneration. Drug delivery via the meningeal lymphatic pathway, such as using the biomimetic nanocomposite biomimetic liposome-curcumin, has been shown to enhance therapeutic outcomes in PD models [259]. Recent work has further suggested that repairing damaged MLVs could represent a novel therapeutic strategy for PD. For example, Cu₂ − xSe nanoparticles were found not only to promote lymphatic vessel growth and development, but also to restore the drainage function of impaired MLVs, thereby improving the transport of immune cells and macromolecules [81]. Nevertheless, the precise mechanisms by which the meningeal immune system contributes to PD pathogenesis remain incompletely understood, and direct evidence in human PD is still lacking. Continued investigation will be essential to clarify these mechanisms and translate lymphatic-targeted interventions into clinical therapies.

Clinically, olfactory dysfunction is recognized as an early symptom of PD, a feature that parallels observations in AD [260]. Studies have also shown that lymphatic vessels located behind the eye connect to the dCLNs via the optic nerve sheath, following a drainage route similar to that of MLVs and subject to regulation by lymphangiogenic factors such as VEGF-C [49]. Given that α-syn pathology is present in the optic nerve and that the glymphatic system is known to clear α-syn via drainage to deep cervical lymph nodes, this pathway may participate in the clearance of neurotoxic proteins such as α-syn, potentially influencing the pathological progression of PD.

## Construction of a clinical assessment and biomarker framework

Precise clinical intervention begins with precise clinical assessment. In the context of addressing the dynamic and intricate brain–lymphatic network, which includes the glymphatic system, MLVs, as well as the brain–eye and the brain–nose drainage pathways, a comprehensive, multimodal, and multidimensional approach is necessary, encompassing the structural, functional, and molecular levels.

### Multimodal neuroimaging assessment techniques

The past decade has witnessed remarkable advances in neuroimaging technologies, enabling in vivo, non‐invasive visualization of CSF flow, meningeal lymphatic drainage, and metabolic clearance processes within the CNS. Such advances provide the foundation for clinical assessment of the brain‐lymphatic network.

#### MRI and DCE‐MRI

DCE-MRI, typically performed via intrathecal contrast injection, is widely used as a tracer-based reference method for assessing glymphatic and meningeal lymphatic dynamics [261]. Using intrathecal gadodiamide injection, studies have assessed clearance at multiple time points (4.5 h, 15 h, and 39 h post‑injection), with peak enhancement typically observed at 15 h and residual signal still detectable at 39 h [262]. In older adults, both glymphatic and putative MLV clearance are significantly delayed compared with younger individuals [262]. High-resolution 3D T2‑fluid‑attenuated inversion recovery (3D T2‑FLAIR) has been shown to be superior to 3D T2‑weighted imaging for displaying human MLVs, particularly when combined with optimized sequences such as black‑blood or dedicated dural‑lymphatic imaging protocols [263]. However, direct assessment of fine MLV morphology remains inherently limited by the spatial resolution, as the typical diameter of lymphatic vessels (~ 0.1 mm or less) is an order of magnitude smaller than the resolution of conventional MRI (~ 1 mm) [264]. Beyond intrathecal DCE-MRI, several non-contrast or indirectly contrast-based MRI techniques provide complementary information. DTI-ALPS yields an ALPS index linked to cognitive decline, AD and PD [265]. Ultrafast functional MRI with EEG quantifies coupling of EEG, blood oxygen level-dependent (BOLD), and CSF signaling and slow oscillations that drive macroscopic CSF waves during deep sleep [194]. Quantitative assessment of enlarged PVS on high-resolution T2-weighted images serves as a structural marker of impaired interstitial and perivascular clearance [266, 267], and dynamic diffusion‑weighted/DTI methods—including CSF‑spatiotemporal rhythmic events and motion‑like approaches—probe pulsatile fluid motion along PVS [268, 269]. Specialized 3D-FLAIR, black‑blood, and dural‑lymphatic imaging sequences may improve visualization of the parasagittal dura, perivenous cysts, and dural lymphatic vessel-related structures [11, 263, 270, 271].

#### PET

PET imaging provides a molecular complement to the structural and dynamic metrics of MRI, permitting direct linkage between lymphatic clearance function and neuropathological burden [272]. For example, using Aβ-binding tracers (such as ^1^⁸F-AV45 or ^11^C-PiB), one can map amyloid deposition in vivo and correlate it spatially and temporally with drainage function [273]. Research shows that in AD models or patients with impaired meningeal lymphatic function, Aβ signals concentrate in brain regions such as the frontal cortex and olfactory cortex, which are key drainage “bottlenecks”, reflecting the non-random accumulation of pathology in poorly drained territories [274, 275]. This spatial overlap between protein accumulation and drainage deficiency supports the mechanistic importance of lymphatic dysfunction in disease progression [163]. Consequently, PET combined with drainage-mapping MRI creates a powerful multimodal approach for mechanistic mapping and potentially patient stratification in neurodegenerative disease [276].

#### Near‐infrared (NIR) fluorescence imaging

Although still largely preclinical, NIR fluorescence imaging offers a highly sensitive, real-time optical modality to track CSF/ISF fluid dynamics via safe fluorescent probes. In animal models, NIR imaging, especially in the NIR-II window, has been used to visualize CSF flow along perivascular pathways and through meningeal lymphatics in real time [277]. Recent studies have developed engineered NIR‑II nanoprobes to dynamically track glymphatic influx, efflux, and brain parenchymal clearance, revealing that long‑term low‑dose dexmedetomidine enhances glymphatic function and rescues sleep deprivation‑induced dysfunction [278].

#### Fundus imaging and brain–eye lymphatic assessment

The retina and optic nerve constitute an extension of the brain with unique accessibility, making them a promising peripheral window into CNS lymphatic function. Using ocular imaging modalities such as optical coherence tomography (OCT) and OCT-angiography, investigators have observed that AD patients often exhibit thinning of the retinal nerve fiber layer and ganglion cell layer, which correlate with intracerebral Aβ burden and cognitive decline [18, 279]. Moreover, recent work has shown that Aβ produced in the brain can be transported along optic pathways to the eye, leading to amyloid deposition in retinal tissues and subsequent neurodegeneration in AD [18]. These findings suggest that the impaired brain–eye lymphatic drainage may result in reduced removal of retinal metabolic waste and contribute to retinal neurodegeneration. Given its non‐invasive, repeatable, and quantifiable nature, fundus imaging has significant promise as a large‐scale screening tool for central lymphatic dysfunction or early AD pathology via retinal biomarkers [280].

#### Nasopharyngeal MRI and the brain–nose pathway

High-resolution, water-sensitive MRI focused on the cribriform plate and olfactory bulb region can visualize the anatomy of the brain–nose drainage channel, through which CSF exits the cranium along olfactory nerve bundles. Recent advances have refined this approach: intravenous administration of gadolinium-based contrast agents enables dynamic assessment of fluid distribution and drainage in human olfactory regions [281]. Moreover, comparative imaging studies have revealed distinct glymphatic–lymphatic coupling dynamics between the nasal mucosa and the parasagittal dura, highlighting regional heterogeneity in CSF clearance mechanisms [282]. Structural alterations in the olfactory region, such as age-related mucosal thickening or cribriform plate obstruction, may indicate impaired clearance via this route [39]. Quantification of CSF flow or contrast tracer movement along the nasal-olfactory pathway provides a complementary, non-invasive assessment of this auxiliary drainage corridor. Although direct human evidence remains limited, animal and cadaveric studies suggest that a substantial portion of CSF outflow occurs through the cribriform/nasal route, underscoring its potential relevance in CNS waste clearance [39]. Notably, a recent study directly visualized CSF and ISF drainage through lymphatic structures surrounding the human middle meningeal artery, providing direct evidence for an extracranial CSF outflow pathway [32]. Future development of dedicated MRI protocols, including contrast-enhanced cisternography or targeted nasal cavity contrast instillation, may further improve the detection of brain–nose drainage efficiency or obstructions in patients with suspected central lymphatic dysfunction.

#### Magnetoencephalography (MEG) and EEG

While MEG and EEG inherently measure neural electrical activity rather than fluid dynamics, they provide valuable functional correlates of lymphatic activity. It is now well established that the glymphatic system is maximally active during slow‐wave sleep (in particular, deep non-REM sleep) when low-frequency oscillations predominate [283, 284]. Slow-wave activity on EEG correlates with the convective clearance of metabolites from the brain interstitium, whereas disturbances in slow-wave sleep are associated with reduced glymphatic flow [117]. Indeed, patients with AD or sleep-architecture disturbances show reduced slow-wave amplitude and spindle density, which may reflect impaired nightly clearance activity [285]. MEG/EEG, by capturing these oscillatory sleep dynamics, thus provide an indirect but functionally relevant index of glymphatic function. Notably, recent human studies combining EEG with MRI have demonstrated that sleep-related changes in BOLD signal fluctuation amplitude follow a sensory-association cortical hierarchy and correlate with inter-individual differences in slow-wave activity, a marker of sleep pressure [286]. Collectively, although MEG/EEG do not measure fluid flow directly, they integrate the neurophysiological correlates of drainage function, especially relevant when assessing the impact of sleep or neuromodulation interventions on waste clearance.

**Fig. 7:**
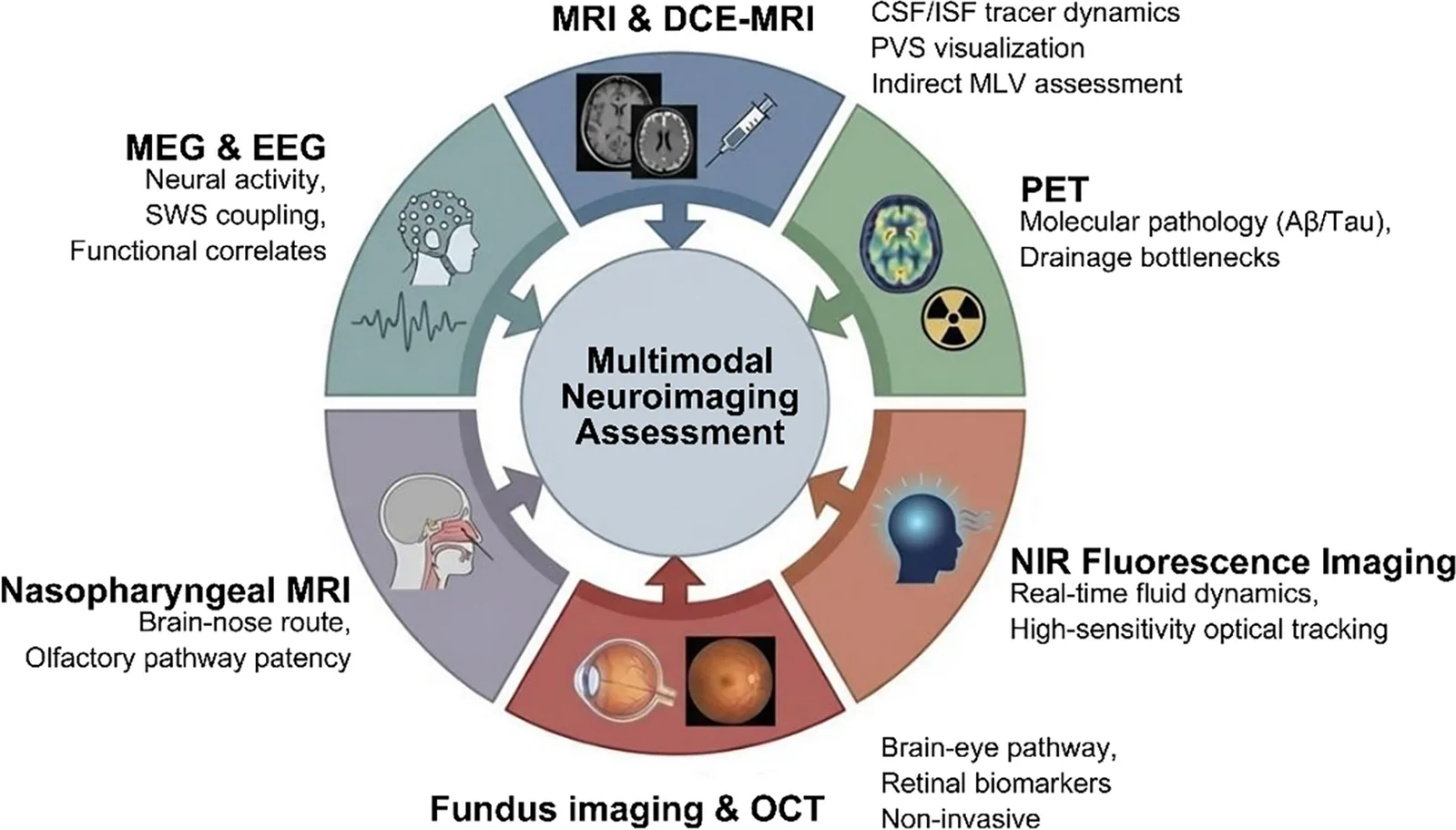
The multimodal neuroimaging assessment techniques

Taken together, these imaging modalities provide complementary structural, functional, and molecular insights into the brain's lymphatic system, forming the basis for a multidimensional assessment (Table 1 and Fig. 7).

**Table 1:** Comparison of multimodal neuroimaging assessments for the brain lymphatic system

Imaging modality	Mechanism /Target	Application focus	Key advantages	Limitations	Current stage
MRI/DCE-MRI	Track paramagnetic contrast kinetics via CSF influx, ISF exchange, and lymphatic efflux	Quantitative assessment of glymphatic dynamics and vessel integrity	Gold standard for in-vivo drainage mapping; quantitative, reproducible, longitudinal imaging; no ionizing radiation	Require invasive tracer/contrast administration; limited spatial–temporal resolution; motion artifacts	Early human studies
PET (Aβ tracers)	Detects Aβ deposition at drainage bottlenecks	Correlates impaired clearance with regional Aβ accumulation	Combines molecular specificity with anatomical precision	Radiation exposure; costly; limited availability	Established for Aβ; exploratory for drainage
NIR-II	Optical tracking of tracer flow in CSF/ISF and lymphatic vessels	Preclinical visualization of real-time glymphatic flow	High sensitivity; real-time dynamic imaging	Limited to animal studies; shallow penetration in humans	Preclinical only
Fundus/OCT imaging	Detects retinal thinning and microvascular changes linked to Aβ	Non-invasive proxy of CNS drainage dysfunction	Rapid, accessible biomarker for neurodegeneration	Indirect measurement; low disease specificity	Early human observational studies
Nasopharyngeal MRI	Visualizes CSF drainage along the olfactory–nasal lymphatic route	Structural mapping of “brain–nose” outflow	Complements DCE-MRI; region-specific insights	Technically demanding; small field of view	Preclinical/early human studies
EEG/MEG	Measures slow-wave activity linked to glymphatic pulsation	Functional assessment of sleep-driven clearance	Non-invasive, repeatable, and low-cost	Indirect; influenced by sleep quality and arousal state	Human physiological studies; indirect

### Biomarkers: the molecular perspective via liquid biopsy

While imaging outlines the “road map” of the brain lymphatic system, liquid biopsy offers the “lab report” of its operational efficiency. Through analysis of CSF and blood biomarkers, one can infer the molecular status of brain–periphery material exchange and waste-clearance efficacy.

#### CSF biomarkers

CSF directly reflects the brain extracellular metabolism, making it ideal for assessing clearance and neurodegeneration. Decreased Aβ₄₂ and increased p-tau217 in the CSF indicate production–clearance imbalances in AD [287]. Elevated glial fibrillary acidic protein (GFAP) is a marker of astrocytic activation, which is in line with experimental data linking AQP4 depolarization to glymphatic failure [288]. Neurofilament light chain serves as a sensitive fluid biomarker of neuroaxonal injury, reflecting the degree of ongoing neurodegeneration [289]. Together, these markers reveal waste accumulation and neurodegeneration.

#### Plasma (or Blood) biomarkers

Blood biomarkers offer clear clinical advantages—they are minimally invasive, repeatable, and scalable for population screening. Plasma p-tau217 and Aβ_42_/Aβ_40_ ratio closely mirror CSF accuracy in AD diagnosis [290]. Genetic polymorphisms in *AQP4* modulate glymphatic function and influence the susceptibility to AD, PD, and sleep disorders [291–293]. These variants may alter AQP4 localization or water transport, affecting baseline clearance efficiency. Moreover, brain-derived metabolites, exosomal microRNAs, and vesicles traveling via nasal–cervical lymphatics are detectable in blood or nasal mucosa [39, 294], providing promising non-invasive markers of impaired cribriform and cervical lymphatic drainage.

#### Inflammation–immune biomarkers

MLVs provide a conduit between the CNS and the peripheral immune system. Accordingly, immune markers at this interface can inform on brain lymphatic status. For example, the polarization state of meningeal macrophages, assessable via markers such as CD163 and CD206, and the ratio of anti‐inflammatory to pro‐inflammatory cytokines (e.g. IL-10/TNF-α) in the CSF or meningeal fluid, reflect the immune milieu of the meninges. Impaired lymphatic drainage can lead to retention of immune cells and cytokines in the meningeal compartment, disrupting immune homeostasis [214]. A relative shift toward pro-inflammatory profiles or an accumulation of central immune cells in peripheral blood may signal stagnation in meningeal lymph outflow. Tracking such immune biomarkers, such as elevated CSF levels of soluble T-cell receptors or cytokine panels, could thus serve as an indirect gauge of meningeal lymphatic function or dysfunction.

**Fig. 8:**
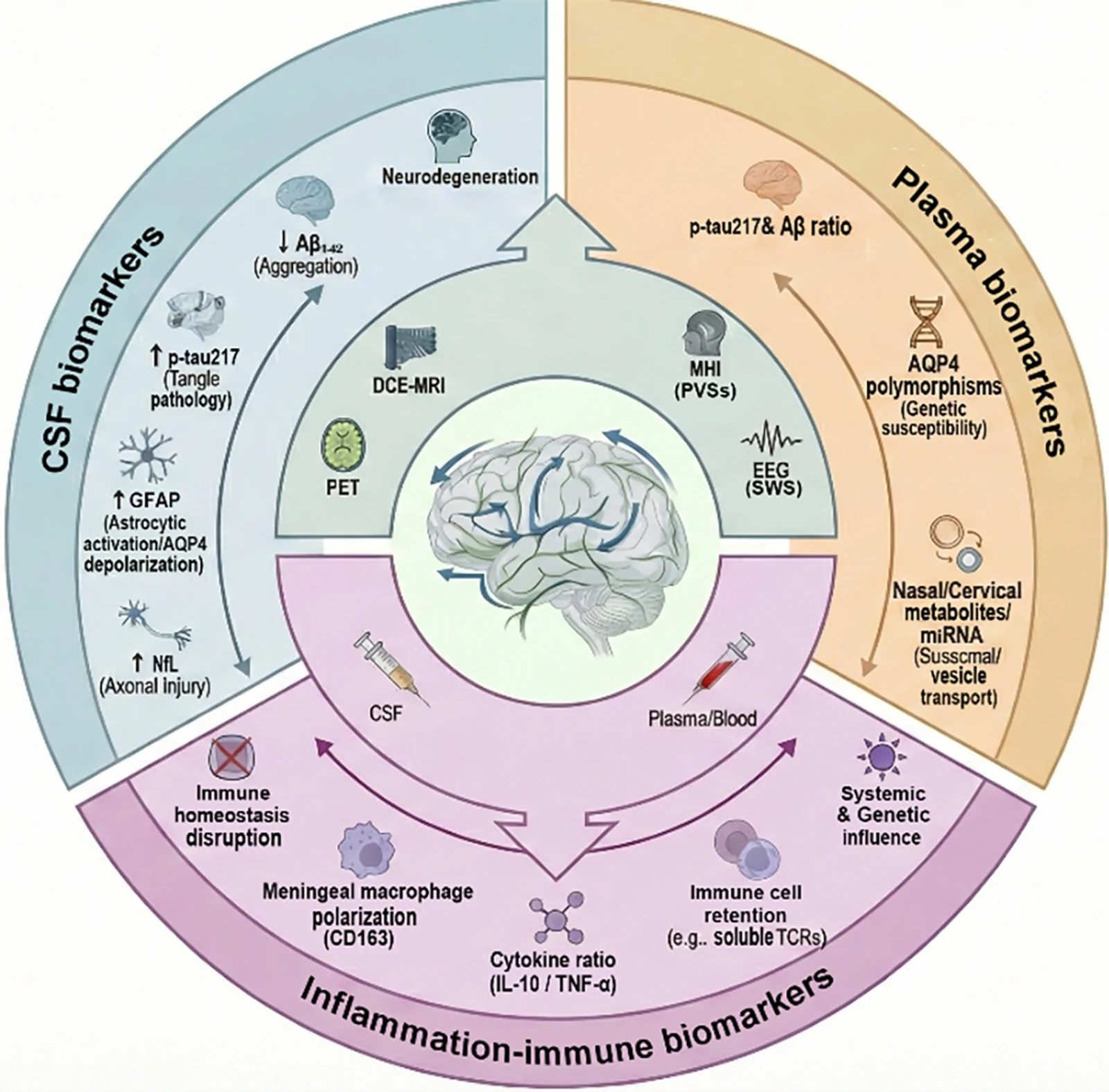
The biomarkers for CNS lymphatic evaluation

In summary, construction of a clinical assessment and biomarker framework for the brain's lymphatic network is now feasible, thanks to advances in imaging and molecular assays. The ultimate goal is to integrate imaging-derived structural/functional data with liquid-biopsy-derived molecular data into a 3D ("structure–function–molecule") assessment model for individual patients. For instance, a patient might exhibit delayed meningeal drainage on DCE-MRI, elevated Aβ deposition in the same region on PET, and a high-risk *AQP4* genotype from plasma. Another might show enlarged PVS on MRI and high CSF level of GFAP, indicating an "astroglial/glymphatic failure" endotype. Such an integrated model allows for early diagnosis, subtype classification, and monitoring of therapeutic responses by tailoring interventions to an individual's specific clearance deficits. The combination of high-resolution dynamic imaging modalities with liquid-biopsy biomarker panels therefore provides a refined, mechanistic view of clearance pathways, structural integrity, functional dynamics, and immune status within the CNS lymphatic system, laying the groundwork for precision therapeutic interventions (Table 2 and Fig. 8).

**Table 2:** Major CSF and blood-based biomarkers reflecting CNS lymphatic and clearance function

Biomarker	Source	Pathophysiological role	Diagnostic value/Relevance	Limitations	Validation
Aβ_42_	CSF	Reflects amyloid production vs clearance imbalance	Decreased in AD; early marker of impaired clearance	Non-specific for disease stage	Clinical AD marker; indirect
p-tau217	CSF/ Plasma	Marker of tau hyperphosphorylation	Elevated in AD; correlates with tau PET	May overlap with other tauopathies	Strong human evidence
GFAP	CSF/ Plasma	Astrocytic activation due to AQP4 depolarization	Indicates early astroglial response and glymphatic failure	Affected by diverse astrocytic insults	Emerging glymphatic marker
NfL	CSF/ Plasma	Axonal injury and neuronal degeneration	Elevated with clearance failure and neurotoxicity	Lacks regional specificity	Clinical injury marker
*AQP4* polymorphisms	Genomic DNA	Modulate AQP4 localization and glymphatic capacity	Genetic susceptibility factor for AD, PD, and sleep disorders	Requires large-scale validation	Association; needs validation
Brain-derived microRNAs/exosomes	Blood/ Nasal mucosa	Reflect drainage through cribriform and cervical lymphatics	Potential non-invasive clearance biomarkers	Exploratory; low standardization	Exploratory

## Precision therapeutic strategies based on assessment

With the comprehensive assessments described above, we can now develop personalized, multi-layered treatment strategies aimed at restoring or enhancing the brain lymphatic system function. The core goal is to improve clearance of metabolic waste, restore CNS homeostasis, and mitigate downstream neurodegeneration and inflammation.

### Lifestyle and physical interventions: activating lymphatic drivers

#### Optimizing sleep

Sleep quality directly influences the glymphatic clearance. Slow-wave sleep in particular is a critical period for glymphatic activity [295]. Thus, improving sleep architecture is foundational for patients with clearance impairments. Interventions such as cognitive behavioral therapy for insomnia, treatment of obstructive sleep apnea, judicious use of sleep aids or chronotherapy, and management of circadian rhythm disorders, can all enhance the duration and the intensity of slow-wave sleep. Ensuring adequate deep sleep effectively provides the nightly “housekeeping” interval during which the glymphatic/meningeal system flushes waste from the brain [74]. Indeed, enhancing slow-wave sleep, for example, through acoustic stimulation or orexin antagonists, has been proposed as a therapeutic strategy to augment glymphatic clearance in AD [296].

#### Sleep positioning

Experimental evidence indicates that lateral sleeping positions, especially the right lateral decubitus posture, promote more efficient CSF–ISF exchange compared with supine or prone positions [297]. In rodent studies, glymphatic transport and Aβ clearance were significantly faster when animals slept on their sides versus on their backs or stomachs [298]. However, this finding has not been replicated in humans, and no clinical evidence currently supports positional recommendations for glymphatic enhancement or AD prevention in patients.

#### Low-intensity transcranial focused ultrasound (FUS)

Safe, low-intensity FUS can modulate astrocytic AQP4 activity and enhance glymphatic flow [299]. In rodents, FUS accelerates tracer clearance without disrupting the BBB, likely via mechanical vibration or TRPV4-calmodulin signaling. It is being explored as a repeatable “glymphatic pump” for early AD.

#### *Breathing techniques and yoga*

Slow, diaphragmatic breathing and yoga postures may improve the lymphatic return by modulating intrathoracic pressure and increasing vagal tone [300, 301]. Gentle inversions transiently raise CSF pressure, facilitating perivascular exchange.

#### Long-term physical exercise

Beyond general recommendations for physical activity, recent longitudinal MRI studies in humans have begun to link structured exercise directly to brain lymphatic function. In a 12-week intervention trial, long-term aerobic exercise preceded by an acute single-bout session increased contrast enhancement along putative MLVs on DCE-MRI and was associated with improved cognitive performance and stress profiles in middle-aged adults [302]. Both single-bout and chronic training paradigms facilitated meningeal lymphatic flow, providing the first in-vivo evidence in humans that sustained exercise can upregulate glymphatic/lymphatic transport. Consistently, animal studies demonstrated that long-term treadmill exercise enhances MLV plasticity and drainage by downregulating the astrocyte-related EAF2–p53–TSP-1 pathway, thereby reducing Aβ deposition and improving cognitive function in AD mice [303], while a companion study showed that the cognitive benefits of voluntary exercise in an AD mouse model depend critically on the presence and the proper polarization of astrocytic AQP4 for effective glymphatic clearance [304]. These findings strengthen the rationale for prescribing structured aerobic exercise as a cornerstone lifestyle intervention in individuals at risk for AD or small-vessel disease.

#### Non-invasive mechanical stimulation of cervical lymphatics

A recent study showed that a servo-controlled external device applying intermittent, low-amplitude mechanical compression over the superficial cervical lymphatic vessels can robustly augment CSF drainage in aged mice [42]. Short daily sessions approximately doubled CSF outflow and ameliorated age-related drainage deficits without impairing the intrinsic contractility of collecting lymphatics. This proof-of-concept suggests that wearable cervical-lymphatic “actuators” could become a practical adjunct to enhance brain waste clearance in patients who are not candidates for invasive procedures.

#### Multisensory gamma stimulation

Another emerging non-pharmacological strategy is the multisensory 40 Hz gamma stimulation. In an AD mouse model, combined visual and auditory 40 Hz stimulation entrained cortical gamma oscillations, improved glymphatic–meningeal clearance of Aβ, reduced plaque burden and partially rescued cognitive deficits [305]. These data align with the sleep literature indicating that synchronized low-frequency oscillations and spindles facilitate CSF pulsation; targeted modulation of network rhythms may therefore represent an indirect way to “drive” the lymphatic pumping apparatus in patients with disturbed sleep or network dysfunction.

### Pharmacological interventions: targeting key nodes

#### Enhancing AQP4 function

Given the central role of AQP4 depolarization as a shared pathological node, interventions aimed at rescuing its polarized localization represent high-priority avenues. These interventions include enhancing α-syntrophin scaffolding [65], stabilizing the dystrophin-associated complex [69], optimizing sleep-dependent circadian regulation [70, 74], etc. Critically, these approaches must be distinguished from global AQP4 upregulation, which lacks functional benefit without concurrent restoration of polarity [166, 167]. Although no AQP4-targeting drug is yet in clinical trials for neurodegenerative disease, ongoing research on AQP4 modulators (and even gene therapy approaches) may yield candidates for boosting glymphatic function in patients.

#### Modulating vascular (arterial) dynamics

Arterial pulsation is a primary driver of glymphatic CSF inflow into periarterial spaces [214]. Thus, treatments that optimize the arterial pulsatility or hemodynamic oscillations might improve glymphatic perfusion. Examples include low-dose vasoactive agents such as certain β-adrenergic blockers or nitric oxide donors, which can subtly enhance arterial compliance and rhythmicity. By promoting a healthier cardiac pulsation wave transmitted to cerebral arterioles, these drugs could amplify the “perivascular pumping” of CSF [214, 306]. Preclinical studies have shown, for instance, that augmenting cardiac stroke volume or arterial pulse amplitude can increase the glymphatic influx, whereas reducing pulsatility diminishes the CSF–ISF exchange. In practice, careful management of blood pressure and avoidance of arterial stiffening agents are advisable. In patients with low cardiac output or stiff arteries, addressing those issues through antihypertensives, exercise, etc., may indirectly restore some glymphatic function. This represents a novel hybrid vascular-lymphatic therapeutic axis: improving blood vessel health to aid brain waste clearance.

#### Expanding/Promoting MLVs

A particularly exciting approach is the use of lymphangiogenic factors to enhance the structure and function of meningeal lymphatics. VEGF-C is a key molecule that stimulates lymphatic vessel growth and remodeling. In aging AD mouse models, administration of VEGF-C (e.g., via viral overexpression or injection) led to enlargement of MLVs, increased CSF drainage to cervical nodes, and consequent reduction in amyloid and tau accumulation [214]. Notably, one study showed that treatment of old APP/PS1 mice with VEGF-C not only improved lymphatic clearance but, when combined with anti-Aβ immunotherapy, dramatically enhanced plaque clearance and cognitive outcomes compared to immunotherapy alone [170, 209]. These findings suggest that structurally “boosting” the lymphatic network can potentiate the effects of other treatments. Translating this approach to humans might involve intrathecal or intranasal delivery of recombinant VEGF-C or small-molecule VEGF-C agonists to safely induce lymphatic vessel growth in the meninges. Caution is needed, as excessive angiogenesis could have side effects, but targeted lymphatic enhancement could be a disease-modifying strategy for conditions like AD.

#### Managing vascular and metabolic comorbidities

 Because cerebral small vessel disease, hypertension, diabetes mellitus, and systemic inflammation all contribute to impaired clearance and cerebrovascular dysfunction, aggressive management of these conditions is essential for creating a healthy substrate on which lymphatic therapies can act. Treating hypertension (to reduce arterial stiffness), improving glycemic control, and lowering systemic inflammation can each remove impediments to glymphatic flow [306]. For example, chronic hypertension is known to reduce the glymphatic clearance efficiency by stiffening arterial walls and altering PVS; antihypertensives might reverse some of these effects. Similarly, diabetes-linked microvascular damage can impair ISF drainage, so antidiabetic medications and lifestyle changes may indirectly bolster the glymphatic system. In essence, optimizing the overall cardiovascular and metabolic health is a foundational step that complements direct brain lymphatic interventions.

### Emerging therapeutic technologies

#### Intranasal brain–nose pathway targeting

As mentioned above, the olfactory cribriform route serves as both a clearance pathway and a gateway for brain drug delivery. Intranasal therapeutics can enhance lymphatic function and neuroprotection. For instance, intranasal borneol, a natural terpene, accelerated Aβ clearance and reduced amyloid burden by enhancing meningeal lymphatic flow in rodents [307]. Additionally, anti-inflammatory agents and insulin are currently under investigation for their potential CNS effects when delivered via perineural routes [159, 161]. This growing body of evidence underscores the potential of targeting the brain–nose pathway as a dual strategy: it not only facilitates the delivery of therapeutic agents, but also promotes the efficient removal of metabolic waste. With advancements in intranasal technologies, this approach holds increasing promise for the treatment of neurological disorders.

#### Bioelectronic medicine

Neuromodulation provides a novel approach to regulate brain lymphatic function. Vagus nerve or cervical sympathetic stimulation can modulate the meningeal lymphatic tone and CSF dynamics through autonomic control. Vagus nerve stimulation, already used for epilepsy and depression, enhances parasympathetic activity, promoting vascular dilation and glymphatic flow [308]. In rodents, vagus nerve stimulation increases CSF tracer influx and waste clearance [308]. Similarly, TMS and related methods may influence sleep oscillations and glial activity tied to glymphatic function. Though still experimental, such bioelectronic strategies may one day “pump” the brain’s cleansing system to combat neurodegeneration.

#### Ultrasound combined with microbubbles

Beyond low-intensity FUS alone, ultrasound can be combined with intravenously administered microbubbles to more strongly modulate BBB permeability and glymphatic transport. In mice, FUS applied over the olfactory bulb or cisterna magna in the presence of microbubbles increased the perivascular movement of albumin and other tracers, thereby enhancing both influx and efflux through glymphatic pathways under optimized parameters [309]. However, this approach is highly parameter-dependent, and repeated or high-intensity exposure may increase BBB permeability, induce neuroinflammation or microglial activation, and potentially cause tissue injury. Therefore, its therapeutic application requires careful optimization of ultrasound power, exposure duration, duty cycle, and microbubble dose, as well as rigorous safety validation. This approach may synergize with intranasal or intracisternal drug delivery by mechanically modulating perivascular fluid movement to improve drug distribution and waste clearance.

#### Deep cervical lymphovenous anastomosis

**Fig. 9:**
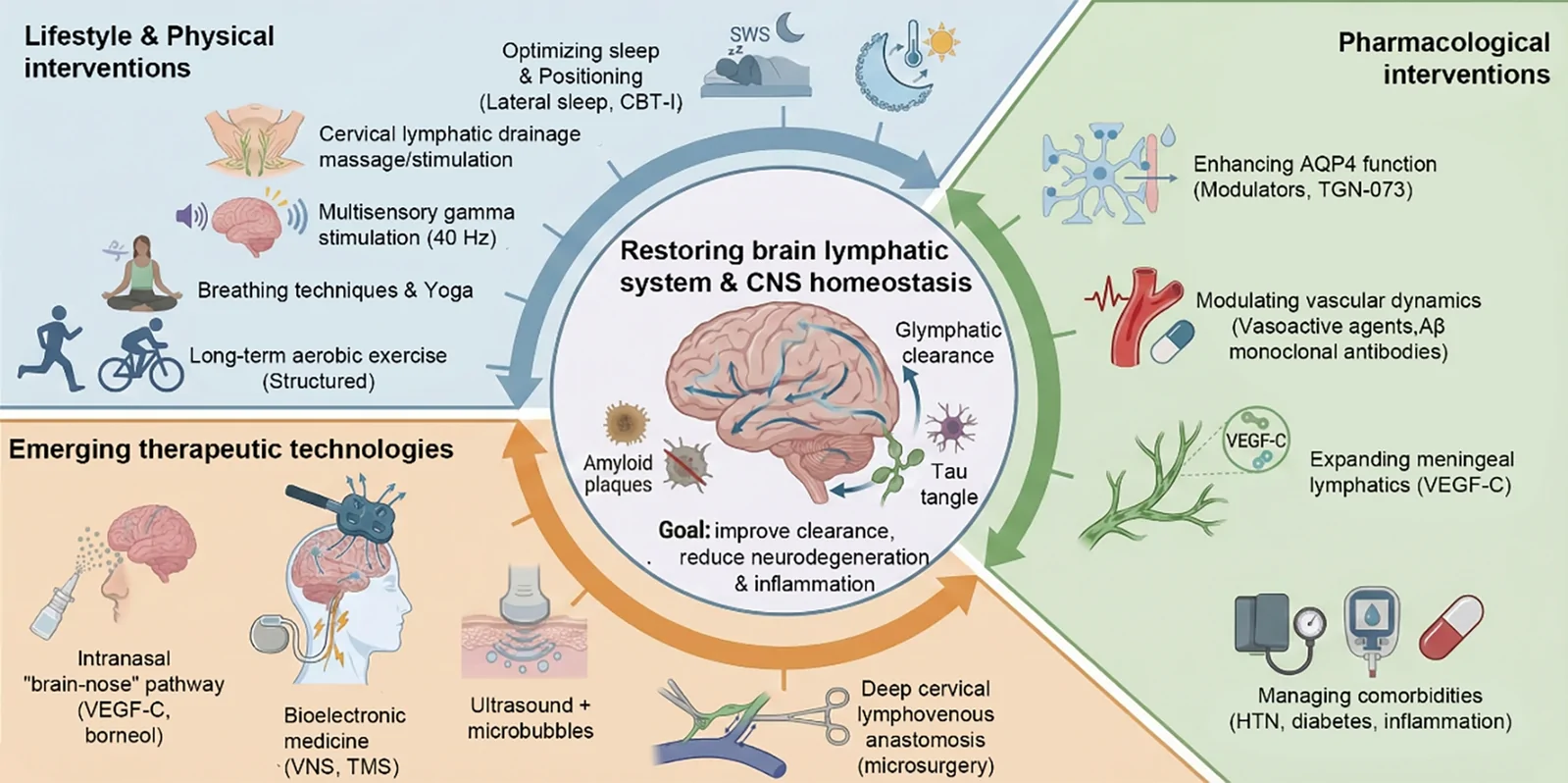
Therapeutic technologies for rescuing CNS lymphatic function

 For advanced or refractory cases, microsurgical reconstruction of cervical lymphatic outflow is being explored. A recent prospective cohort study reported that deep cervical lymphovenous anastomosis, in which collecting lymphatic vessels are anastomosed to nearby veins, was technically feasible in AD patients and could restore the patency of obstructed cervical lymphatic channels [310]. Early follow-up suggested improvement in CSF/lymphatic drainage parameters and stabilization of cognitive decline in some patients, although larger controlled trials are needed to confirm efficacy and long-term safety. This line of work illustrates how principles from peripheral lymphedema surgery may be adapted to target central lymphatic dysfunction (Table 3 and Fig. 9).

**Table 3:** Overview of lifestyle, physical, and technological interventions targeting CNS lymphatic function

Intervention	Mechanism/Target	Application mode	Key outcomes	Limitations	Current stage
Cervical lymphatic drainage massage	Enhances deep cervical lymph flow	Manual therapy; gentle neck massage	May promote meningeal outflow, relieve congestion	Anecdotal evidence; lacks controlled studies	Anecdotal/early clinical
Low-intensity transcranial FUS	Modulates astrocytic AQP4 and TRPV4 signaling	Non-invasive cranial sonication	Accelerates tracer clearance and enhances glymphatic flow	Preclinical stage; optimal parameters uncertain	Preclinical only
Breathing techniques/Yoga	Alters intrathoracic pressure and vagal tone	Slow diaphragmatic breathing, inversion poses	Improves venous/lymphatic return and parasympathetic tone	Limited mechanistic evidence; user-dependent	Indirect human evidence
AQP4 modulators (e.g., TGN-073)	Increase perivascular AQP4 clustering	Pharmacological administration	Enhances CSF–ISF exchange, reduces tau burden	Safety and efficacy not yet established	Preclinical only
Vagus nerve stimulation	Activates parasympathetic pathways controlling CSF flow	Implanted/non-invasive device	Boosts glymphatic influx and waste clearance	Experimental; long-term effects unknown	Preclinical for clearance
Multisensory γ stimulation	Entrains 40Hz cortical rhythms; activates microglia	Non-invasive audiovisual (light & sound)	Reduces plaque burden; accelerates glymphatic clearance	Indirect mechanism; efficacy varies in late-stage	Preclinical/early human cohort
Servo-controlled mechanical stimulation	Intermittent compression of superficial lymphatics	Wearable external neck device ("actuators")	Doubles the CSF outflow (aged mice); ameliorates deficits	Proof-of-concept in mice; device in development	Preclinical proof-of-concept
Deep cervical lymphovenous anastomosis	Bypasses lymphatic obstruction; lowers resistance	Microsurgical reconstruction (lymphatics to veins)	Reduces intracranial fluid burden; cognitive stabilization	Invasive surgery; requires large controlled trials	Early human cohort
FUS + microbubbles	Mechanically "stirs" perivascular fluid	Transcranial FUS + Intravenous microbubbles	Potentially enhances targeted solute transport and BBB permeability under optimized parameters	Possible neuroinflammation or tissue injury; safety requires validation	Preclinical for clearance; early clinical for BBB opening

In summary, research on the brain’s lymphatic system has opened a new frontier in our understanding of CNS homeostasis and disease mechanisms. The multimodal imaging and liquid-biopsy assessment framework described above is a critical bridge between bench science and clinical translation, allowing us to identify specific clearance deficits in individuals. Based on these detailed assessments, clinicians and researchers can now envision patient-specific “drainage phenotypes”, such as an “AQP4 dysfunction” subtype, a “meningeal outflow obstruction” subtype, or a “sleep-dependent impairment” subtype, and tailor interventions across lifestyle, pharmacologic, and technological domains accordingly.

As imaging resolution increases, molecular biomarkers are becoming more specific, targeted therapies are emerging, and the brain’s lymphatic system is poised to become a central therapeutic axis in neurology. With sustained interdisciplinary effort, it appears feasible to delay or even prevent the progression of neurodegenerative, cerebrovascular, and neuroinflammatory brain disorders by restoring the brain’s own cleansing pathways. The coming years will likely see clinical trials of glymphatic modulators, lifestyle prescriptions for sleep and posture to boost clearance, and perhaps implantable devices to rhythmically stimulate lymphatic flow. In conclusion, precision medicine for CNS diseases will increasingly incorporate strategies to monitor and enhance the brain’s lymphatic function, bringing new hope for conditions long deemed intractable.

## Conclusions and future perspectives

The central lymphatic network, including the glymphatic system, MLVs, and auxiliary pathways such as the “brain–nose” and “brain–eye” routes, constitutes a highly coordinated system for intracranial waste clearance and immune surveillance. Recent studies have not only elucidated its fundamental role in maintaining brain homeostasis, but have also established its critical pathological relevance in aging and various neurodegeneration disorders. Dysfunction of this network leads to impaired metabolic waste clearance, abnormal immune-cell trafficking, and persistent neuroinflammation, thereby creating a self-perpetuating pathological cycle. With advances in high-resolution imaging, liquid biopsy technologies, and gene-editing approaches, our understanding of the spatiotemporal dynamics, molecular regulation, and immunological interactions of the central lymphatic network continues to deepen. For instance, loss of AQP4 polarization, attenuation of VEGF-C/VEGFR-3 signaling, and aberrant Piezo1-mediated mechanosensation have emerged as potential therapeutic targets. The development of multimodal imaging tools and biomarker frameworks further provides new opportunities for early clinical diagnosis and evaluation of therapeutic efficacy. Despite these significant advances in basic research, substantial challenges remain in translating this knowledge from experimental discoveries into clinical strategies. Our understanding of the functional heterogeneity of the glymphatic system across different brain regions, cell types, and disease stages remains markedly limited. The efficiency of the glymphatic system varies dynamically across the sleep–wake cycle and among specific brain regions, such as the hippocampus and cortex, yet the precise mechanisms governing these spatiotemporal specificities have not been fully elucidated. Similarly, dorsal and basal MLVs exhibit substantial differences in structure, function, and vulnerability to aging, yet the molecular determinants underlying these distinctions remain unclear. Future research must leverage cutting-edge technologies, including single-cell RNA sequencing, spatial transcriptomics, lineage tracing, and high-resolution in vivo imaging, to construct a fine-grained, cell-level atlas of the central lymphatic network. Particular emphasis should be placed on delineating the specialized communication networks among meningeal lymphatic endothelial cells, fibroblasts, and immune cells. And clarifying how these intercellular interactions are selectively disrupted in distinct pathological contexts such as AD and PD. Such insights will provide a critical foundation for developing interventions tailored to specific cell types and disease stages.

Transforming assessments of central lymphatic function from research tools into reliable clinical diagnostic indicators remains one of the most pressing challenges in the field. Although techniques such as the DTI-ALPS index and DCE-MRI show great promise, substantial variability exists across research centers in image acquisition protocols, post-processing pipelines, and interpretation of results, reflecting a lack of unified and standardized operating procedures. Moreover, the relationships between these imaging-based metrics and emerging CSF or blood biomarkers, such as AQP4 autoantibodies, GFAP, and lymphangiogenic factors, require validation in large-scale, multicenter prospective cohort studies. A key priority for future work is to establish a unified, non-invasive, and reproducible integrated “imaging–liquid biopsy” evaluation framework, including the definition of normal reference ranges and pathological thresholds for each metric and their dynamic changes across disease stages. Such efforts will be essential for enabling central lymphatic function assessment to become a routine clinical tool for early diagnosis, staging, and therapeutic monitoring in neurodegenerative diseases.

Intervention strategies such as VEGF-C protein delivery, AAV-mediated enhancement of VEGFR-3 signaling, regulation of AQP4 polarization, and activation of the mechanosensitive ion channel Piezo1 (e.g., with the agonist Yoda1) have demonstrated substantial therapeutic potential in preclinical animal models by improving lymphatic drainage, promoting the clearance of pathological proteins, and alleviating cognitive deficits. However, major barriers remain in translating these approaches to clinical application. A central concern is long-term safety and off-target effects. For instance, while VEGF-C promotes the regeneration of MLVs, it may also exacerbate pathological angiogenesis or facilitate potential tumor metastasis. Furthermore, it is unclear how systemic or intracerebroventricular administration can achieve specific targeting of MLVs without affecting other tissues. Similarly, activation of the broadly expressed Piezo1 channel may produce unforeseen adverse effects in peripheral organs such as the cardiovascular system. Therefore, future research must prioritize the development of tissue-specific or cell-type-specific delivery systems, including nanocarriers and cell-penetrating peptides, and conduct rigorous dose-optimization and long-term toxicity assessments. These efforts are essential for defining the therapeutic window of emerging interventions and ensuring both efficacy and safety in human applications. The function of the central lymphatic network is profoundly shaped by systemic physiological states, such as sleep, blood pressure, and metabolism, as well as local microenvironmental factors including arterial pulsatility and intracranial pressure. This complexity suggests that single-target pharmacological interventions may yield only limited benefits. Consequently, future therapeutic paradigms must adopt a multimodal and interdisciplinary approach. Such a paradigm requires integrating targeted pharmacological or biological therapies (e.g., VEGF-C) with optimized non-pharmacological interventions, including sleep–wake rhythm modulation, personalized aerobic exercise programs, respiratory training, and even physical therapies such as low-intensity FUS. These combinations may produce synergistic, supra-additive effects. For example, pharmacological enhancement of meningeal lymphatic structure, paired with sleep interventions that maximize nocturnal glymphatic clearance, may achieve therapeutic outcomes greater than the sum of either strategy alone. Equally important is the recognition that a patient’s genetic background (e.g., *APOE4* genotype), age, sex, comorbidities (such as hypertension and diabetes), and lifestyle factors substantially influence their baseline lymphatic function and responsiveness to therapy. Thus, future clinical trial designs must embrace a precision medicine framework, identifying subpopulations most likely to benefit based on lymphatic imaging phenotypes, genomic profiles, and clinical characteristics. Tailoring individualized strategies to restore central lymphatic function will be essential not only for the successful translation of basic discoveries into clinical practice, but also for achieving meaningful prevention and control of neurodegenerative diseases.

## Data Availability

No datasets were generated or analysed during the current study.

## Abbreviations

Aβ: Amyloid-β
ACE: Arachnoid cuff exit
AD: Alzheimer's disease
AKT: Protein kinase B
ALPS: Along the perivascular space
AQP4: Aquaporin-4
AAV: Adeno-associated virus
BBB: Blood-brain barrier
CAA: Cerebral amyloid angiopathy
cDC: Conventional dendritic cell
CLNs: Cervical lymph nodes
CNS: Central nervous system
CSF: Cerebrospinal fluid
CXCR: C-X-C chemokine receptor type
dCLNs: Deep cervical lymph nodes
DCE-MRI: Dynamic contrast-enhanced MRI
DTI-ALPS: Diffusion tensor imaging analysis along the perivascular space
EEG: Electroencephalogram
EFNB2: Ephrin B2
ELS: Ectopic lymphoid follicles
EPHB4: Ephrin type-B receptor 4
ERK: Extracellular signal-regulated kinase
FGF2: Fibroblast growth factor-2
FOXC2: Forkhead box C2
FUS: Focused ultrasound
GFAP: Glial fibrillary acidic protein
GFP: Green fluorescent protein
HGF: Hepatocyte growth factor
IFN-γ: Interferon-gamma
IL: Interleukin
iPSC: Induced pluripotent stem cell
IPAD: Intramural periarterial drainage
ISF: Interstitial fluid
LNs: Lymph nodes
LYVE-1: Lymphatic vessel endothelial hyaluronan receptor-1
MAPK: Mitogen-activated protein kinase
MANF: Mesencephalic astrocyte-derived neurotrophic factor
MEG: Magnetoencephalography
MHC-II: Major histocompatibility complex II
MLVs: Meningeal lymphatic vessels
MRI: Magnetic resonance imaging
NALT: Nasal-associated lymphoid tissue
NIR: Near-infrared
NK: Natural killer
NLVs: Nasal lymphatic vessels
NMO: Neuromyelitis optica
NPLP: Nasopharyngeal lymphatic plexus
NREM: Non-rapid eye movement
OCT: Optical coherence tomography
PBMs: Parenchymal border macrophages
PD: Parkinson's disease
PET: Positron emission tomography
PI3K: Phosphatidylinositol 3-kinase
Prox1: Prospero homeobox protein 1
PVS: Perivascular spaces
RASA1: RAS p21 protein activator 1
REM: Rapid eye movement
SLYM: Subarachnoid lymphatic-like membrane
TBI: Traumatic brain injury
TMS: Transcranial magnetic stimulation
TNF-α: Tumor necrosis factor-α
Tregs: Regulatory T cells
TRPV4: Transient receptor potential vanilloid 4
TZ: Transition zone
VEGF: Vascular endothelial growth factor
α-syn: α-Synuclein
